# Insights into the taxonomic and functional characterization of agricultural crop core rhizobiomes and their potential microbial drivers

**DOI:** 10.1038/s41598-021-89569-7

**Published:** 2021-05-12

**Authors:** Antonio Castellano-Hinojosa, Sarah L. Strauss

**Affiliations:** University of Florida, Institute of Food and Agricultural Sciences, Southwest Florida Research and Education Center, 2685 State Rd 29N, Immokalee, FL 34142 USA

**Keywords:** Agroecology, Microbial ecology, Microbial communities, Environmental microbiology

## Abstract

While our understanding of plant–microbe interactions in the rhizosphere microbiome (rhizobiome) has increased, there is still limited information on which taxa and functions drive these rhizobiome interactions. Focusing on the core rhizobiome (members common to two or more microbial assemblages) of crops may reduce the number of targets for determining these interactions, as they are expected to have greater influence on soil nutrient cycling and plant growth than the rest of the rhizobiome. Here, we examined whether the characterization of a core rhizobiome on the basis of only taxonomic or functional traits rather than the combined analysis of taxonomic and functional traits provides a different assessment of the core rhizobiome of agricultural crops. Sequences of the bacterial 16S rRNA gene from six globally important crops were analyzed using two different approaches in order to identify and characterize the taxonomic and functional core rhizobiome. For all crops examined, we found significant differences in the taxonomic and functional composition between the core rhizobiomes, and different phyla, genera, and predicted microbial functions were dominant depending on the core rhizobiome type. Network analysis indicated potentially important taxa were present in both taxonomic and functional core rhizobiomes. A subset of genera and predicted functions were exclusively or predominately present in only one type of core rhizobiome while others were detected in both core rhizobiomes. These results highlight the necessity of including both taxonomy and function when assessing the core rhizobiome, as this will enhance our understanding of the relationships between microbial taxa and soil health, plant growth, and agricultural sustainability.

## Introduction

The root-rhizosphere interface is a nexus of plant–microbe interactions that assist plant uptake of mineral nutrients or water^1,5^. This interface is a key component for agricultural microbiome engineering as it has been used to maximize microbiome functions in agroecosystems to increase plant nutrient uptake^6,7^ and resistance to biotic and abiotic stresses^1,8,9^. These engineering projects, and the potential for additional manipulation of microbiome functions, makes the examination of these interactions a prerequisite for the future of global agriculture^5,10–13^. In particular, there is increased focus on identifying taxa or components of the rhizosphere microbiome (rhizobiome) that can be utilized to improve agriculture sustainability, however, the number of potential targets in the entire rhizobiome, and the variation between crops, is immense^5,14–17^. Determining and characterizing the “core” rhizobiome (members common to two or more microbial assemblages associated with a habitat^18^) of crops could narrow this search for targets, as this “core” microbiota is hypothesized to have greater influence on soil nutrient cycling and the physiology, growth, and health of the host plant than the rest of the rhizobiome^1–3,5,19^. Yet multiple definitions of the term core rhizobiome have been used across studies to try and identify key microbes based on their presence within a host population, spatial distribution, temporal stability, or contribution to host function and fitness^18,20^.

Despite the differences in definitions, a core rhizobiome in certain plant species has been identified, including *Arabidopsis*^21^, barley^22^, citrus^23^, maize^24^, common bean^25^, cucumber^26^, grapevine^27^, millet^28^, populus^29^, rice^30^, soybean^31^, sugarcane^32^, tomato^33^, and wheat^34–36^. These studies identified operational taxonomic unit (OTUs) that were present in the rhizosphere in a high proportion of the sequencing samples (e.g., > 75% of the samples), but only four of these studies also looked at the functional traits in the core rhizobiome using metagenomic sequencing^23,26,31^ or other tools^28^ such as PICRUSt2^37^ that can predict microbial functions from 16S rRNA sequences. The latter core rhizobiome study used PICRUSt2 to identify a number of Kyoto Encyclopedia of Genes and Genomes (KEGG) orthologs (KOs^38^) that were present in a high proportion of the samples (e.g., > 75% of the samples) but did not identify taxa associated with core functions in the rhizobiome.

However, the description of the core rhizobiome taxa on the basis of taxonomy may provide only a partial characterization of the core rhizobiome^20,39,40^. For example, bacterial diversity in the rhizosphere can differ depending upon soil types^41^ and plant genotypes^42–44^ and can be heavily influenced by abiotic and environmental conditions^19,45,46^. In addition, functional traits rather than taxonomy seem to dominate the recruitment of bacterial communities in the rhizosphere^22,26,31,43,47^. Although previous studies have identified core rhizobiome taxa and rhizobiome functional genes of soybean^31^, cucumber and wheat^26^, millet^28^, and citrus^23^, they did not look at the association between core taxa and functions within the core rhizobiome. Lemanceau et al.^39^ proposed the implementation of a new strategy for the characterization of core rhizobiomes through which not only the “taxonomic core rhizobiome” but also the “functional core rhizobiome” should be identified as potential microbial drivers in a core rhizobiome. However, it is still unclear how, and to what extent, a combined analysis of the taxonomic and functional description of the core rhizobiome rather than a single taxonomic or functional approach could provide additional insights into the identification and characterization of core rhizobiomes. A better characterization of core rhizobiomes and their potentially important microbial drivers is critical not only to increase our understanding of plant–microbe interactions occurring in this interface^2–5^ but also to assess the impact of external forces such as management practices (e.g., crop rotation, tillage, and fertilization) in the rhizosphere microbial community^48^. Improved understanding of microbe-microbe interactions occurring in the rhizosphere can also help identify microbial “hubs,” small numbers of taxa that are strongly interconnected, which are thought to be disproportionally important in shaping microbial communities of plant hosts, and important intermediates between abiotic and host factors^49,50^. In addition, because rhizobiomes often involve taxa with the same or similar functions (that is, functional redundancy^51^), increased knowledge about taxonomic and functional taxa in the core rhizobiome may help to better characterize functional redundancy, as previously shown in the human gut^52^.

Here we present an exploratory effort to identify the bacterial taxa and functions belonging to taxonomic and functional core rhizobiomes of crops. Our study had two main objectives: (1) to determine whether the characterization of a core rhizobiome on the basis of only taxonomic or functional traits rather than the combined analysis of taxonomic and functional traits provides a different description of the core rhizobiome; and (2) to examine potential interactions between taxa belonging to both the taxonomic and functional core rhizobiomes and identify highly interconnected taxa, defined here as “hub taxa.” We hypothesize that: (1) because taxonomic and functional traits seem to drive the recruitment of microbial populations by plants^22,26,39,43,47,51^, the combined analysis of the taxonomic and functional description of the core rhizobiome will lead to a more complete characterization of core rhizobiomes than the use of only a taxonomic or functional approach; and (2) if hypothesis #1 is supported, hub taxa will be identified in both taxonomic and functional core rhizobiomes. To test these hypotheses, we used data from six previously published rhizobiome studies of the globally important crops^53^ rice^30^, wheat^34^, maize^24^, citrus^23^, sugarcane^32^, and tomato^33^, and obtained over 5800 samples and 770 million sequences of the bacterial 16S rRNA gene. In addition, a subset of samples from the rhizosphere of citrus (*C. sinensis; C. paradise; C. reticula;* and *C. grandis*) in Florida (herein citrus #2) was included. For each rhizobiome study, samples were analyzed using two distinct approaches to determine the taxonomic and the functional core rhizobiomes.

## Results

### Bacterial community composition of the taxonomic and functional core rhizobiomes

**Figure 1:**
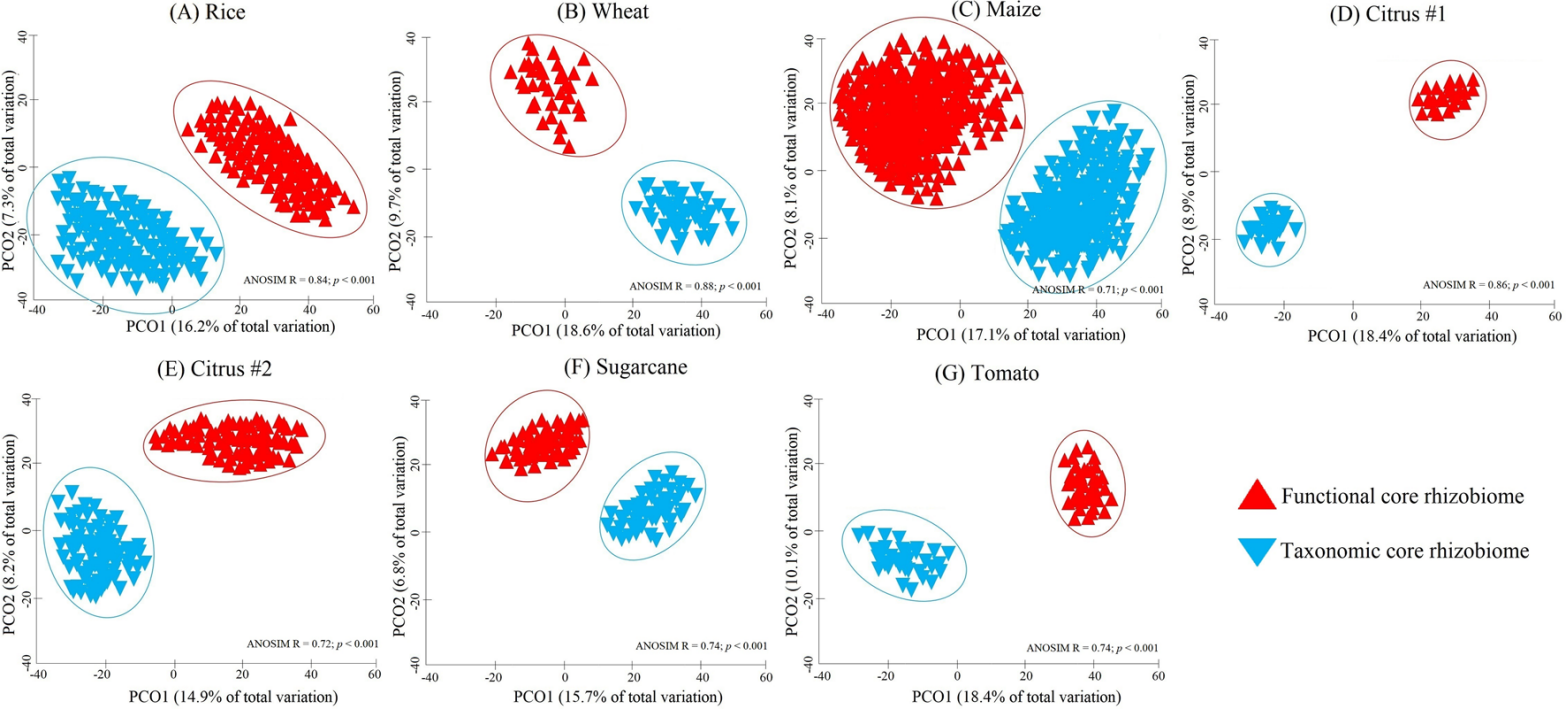
Principal coordinates analysis (PCoA) plots of Bray–Curtis similarities of taxonomic and functional core rhizobiomes calculated on unweighted UniFrac distances for rice (**A**), wheat (**B**), maize (**C**), citrus #1 (**D**), citrus #2 (**E**), sugarcane (**F**), and tomato (**G**). Differences in bacterial community composition between core rhizobiomes were tested by analysis of similarities (ANOSIM), and *p*-values < 0.01 were considered significant. R values showing the extent of bacterial community variation between core rhizobiomes are indicated, and R values close to "1.0" suggest dissimilarity between groups.

For all rhizobiome studies, there were significant differences in the bacterial community composition of the taxonomic and functional core rhizobiomes (*p* < 0.001; Fig. 1A–G). A significantly greater number of ASVs were identified in the functional compared to the taxonomic core rhizobiome for all studies (Table S3). The taxonomic and functional core rhizobiomes comprised, on average, only 0.2–3.3% and 0.9–4.2%, respectively, of all bacterial ASVs observed in the rhizobiome of each crop. However, these taxonomic and functional core ASVs accounted for, on average, 6–12% and 8–18%, respectively, of the relative abundance of all bacteria detected in the rhizobiomes. Number and taxonomic affiliation of shared ASVs between the taxonomic and functional core rhizobiomes for each rhizobiome study are presented in Table S4. Shared taxa represented about 8–15% of the total number of ASVs identified in both core rhizobiomes (Table S4). Phylogenetic trees of the ASVs identified in the taxonomic and functional core rhizobiomes of each crop revealed that there was not a phylogenetic signal associated with core taxonomic and functional ASVs (Fig. S2A–G).

**Figure 2:**
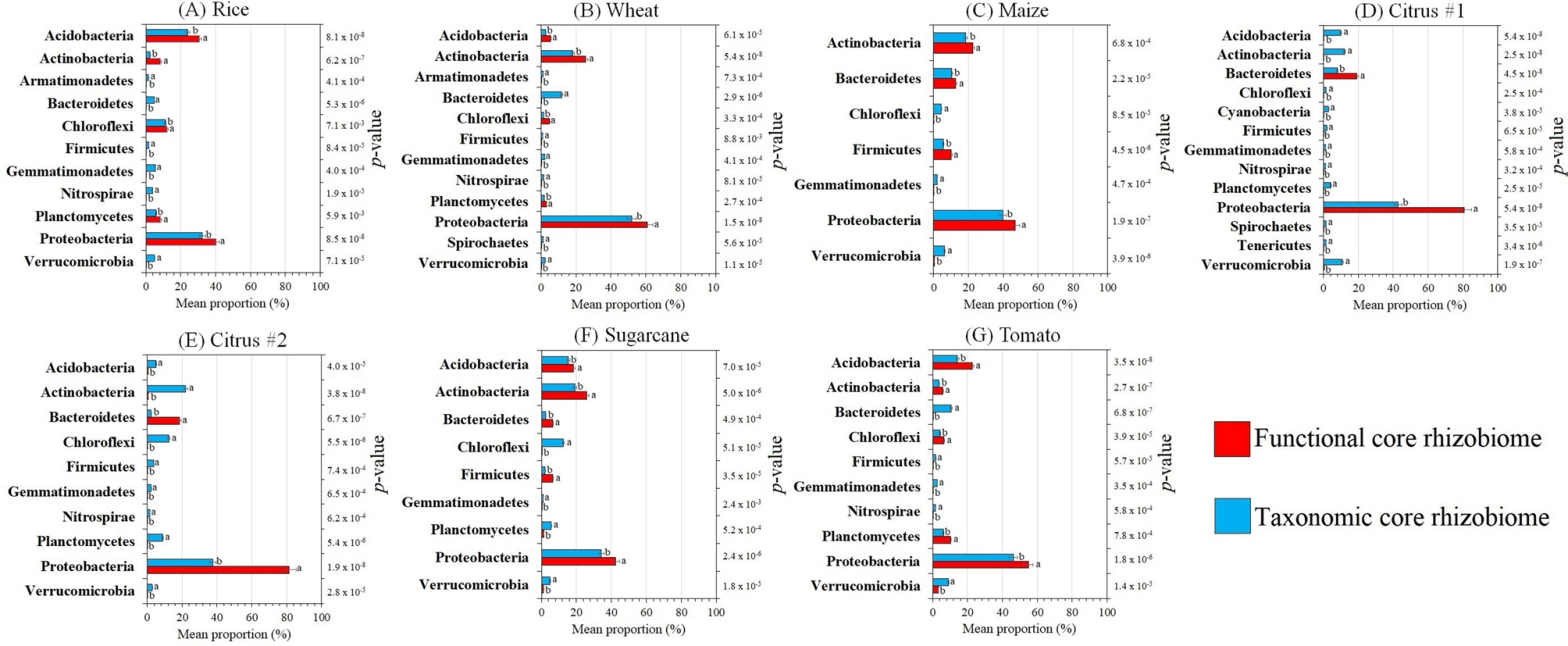
Histograms of phyla significantly different between taxonomic and functional core rhizobiomes according to the Welch’s t-test and Benjamini–Hochberg FDR multiple test correction for rice (**A**), wheat (**B**), maize (**C**), citrus #1 (**D**), citrus #2 (**E**), sugarcane (**F**), and tomato (**G**). The mean proportion (%) of the phyla in each taxonomic or functional core rhizobiome is shown. Letters above the bars indicate significant differences in the relative abundance of each phylum between taxonomic and functional core rhizobiomes, and *p*-values < 0.05 were considered significant. Bars marked with the same letter are not significantly different.

The relative abundances of a subset of the phyla identified in both core rhizobiomes were significantly different (*p* < 0.05) between taxonomic and functional core rhizobiomes for rice (11 phyla), wheat (12 phyla), maize (8 phyla), citrus #1 (13 phyla), citrus #2 (10 phyla), sugarcane (9 phyla), and tomato (10 phyla) (Fig. 2). For rice (Fig. 2A), wheat (Fig. 2B), and tomato (Fig. 2G), Acidobacteria, Actinobacteria, Chloroflexi, Plantomycetes, and Proteobacteria were significantly more abundant in the functional than in the taxonomic core rhizobiomes. For maize (Fig. 2C) and sugarcane (Fig. 2F), functional core rhizobiomes had statistically higher abundances of Actinobacteria, Bacteroidetes, Firmicutes, and Proteobacteria than taxonomic core rhizobiomes. For Citrus #1 (Fig. 2D) and Citrus #2 (Fig. 2E), Bacteroidetes and Proteobacteria were significantly more abundant in functional rather than in the taxonomic core rhizobiomes.

The relative abundances of a subset of the genera identified in the core rhizobiomes were significantly different (*p* < 0.05) between taxonomic and functional core rhizobiomes for rice (15 genera), wheat (14 genera), maize (12 genera), citrus #1 (25 genera), citrus #2 (36 genera), sugarcane (13 genera), and tomato (12 genera) (Fig. S3). For each rhizobiome study, a subset of specific genera was exclusively detected in one type of core rhizobiome (Supplementary Data 1): rice (40 genera), wheat (17 genera), maize (15 genera), citrus #1 (28 genera), citrus #2 (22 genera), sugarcane (15 genera), and tomato (17 genera).

### Predicted functional traits of the taxonomic and functional core rhizobiomes

**Figure 3:**
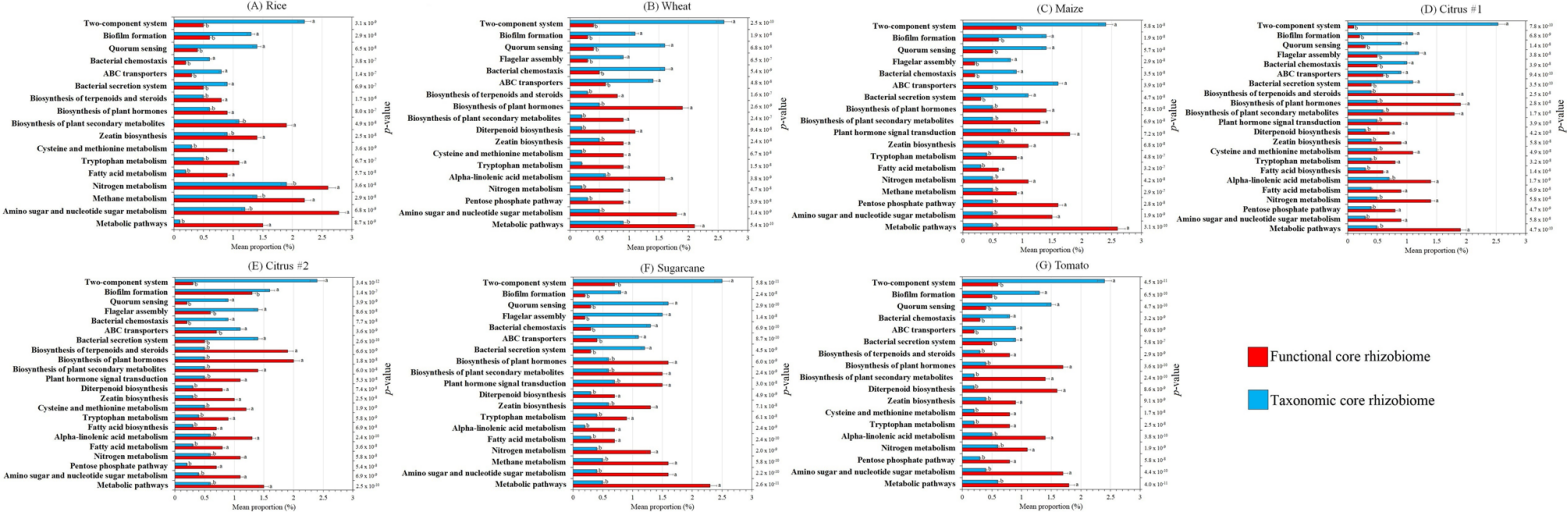
Histograms of predicted pathways significantly different between taxonomic and functional core rhizobiome samples according to the Welch’s t-test and Benjamini–Hochberg FDR multiple test correction for rice (**A**), wheat (**B**), maize (**C**), citrus #1 (**D**), citrus #2 (**E**), sugarcane (**F**), and tomato (**G**). The mean proportion (%) of the predicted pathways in each taxonomic or functional core rhizobiome is shown. Letters above the bars indicate significant differences in the relative abundance of each pathway between taxonomic and functional core rhizobiomes, and *p*-values < 0.05 were considered significant. Bars marked with the same letter are not significantly different.

For all rhizobiome studies, a significantly greater number of pathways and KOs were detected in functional rather than taxonomic core rhizobiomes (Table S5). Significant variations in the functional composition of the core rhizobiome between taxonomic and functional core rhizobiomes were detected both at the pathway and KO levels for all rhizobiome studies (*p* < 0.001; Table 2). A subset of the pathways identified in the core rhizobiomes showed significant differences (*p* < 0.05) in their relative abundance between taxonomic and functional core rhizobiomes for rice (17 pathways), wheat (18 pathways), maize (18 pathways), citrus #1 (22 pathways), citrus #2 (22 pathways), sugarcane (19 pathways), and tomato (18 pathways) (Fig. 3). In particular, pathways assigned to global metabolism (metabolic pathways), carbohydrate metabolism (amino sugar and nucleotide sugar metabolism and pentose phosphate pathways), energy metabolism (methane and nitrogen metabolisms), lipid metabolism (fatty acid and alpha-linolenic acid metabolism), metabolism of other amino acids (fatty acid biosynthesis), amino acid metabolism (tryptophan and cysteine and methionine metabolisms), metabolism of terpenoids and polyketides (zeatin and diterpenoid biosynthesis), signal transduction (plant hormone signal transduction), and chemical structure transformations (biosynthesis of plant secondary metabolites, plant hormones and terpernoids and steroids) were significantly more abundant in functional rather than taxonomic core rhizobiomes (Fig. 3). Pathways involved in membrane transport (bacterial secretion system and ABC transporters), cell motility (bacterial chemotaxis and flagellar assembly), cellular community (quorum sensing and biofilm formation), and signal transduction (two-component system) showed a statistically greater abundance in the taxonomic than functional core rhizobiomes (Fig. 3).

A subset of the KOs identified in the core rhizobiomes showed significant differences (*p* < 0.05) in their relative abundances between taxonomic and functional core rhizobiomes for each crop (Supplementary Data 2) or were exclusively detected in one type of core rhizobiome (Supplementary Data 3).

### Network analysis and hub taxa

**Figure 4:**
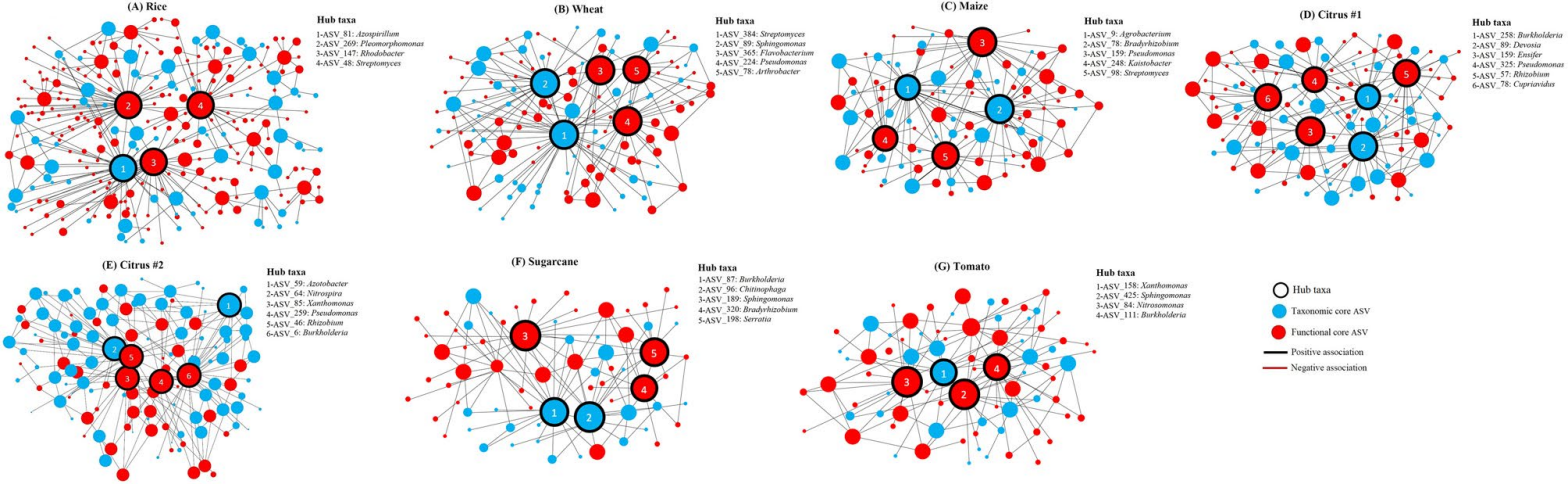
Co-occurrence networks showing significant associations between taxonomic and functional core OTUs belonging to the taxonomic and functional core rhizobiomes, respectively for rice (**A**), wheat (**B**), maize (**C**), citrus #1 (**D**), citrus #2 (**E**), sugarcane (**F**), and tomato (**G**). Node size reflects the node degree (number of neighbours/correlations in the network). Hub taxa are listed and highlighted with black borders in the networks and were identified as per Fig. S4. The full taxonomic affiliation of the hub taxa is presented in Table S6. Network visualization was performed using Cytoscape v3.7.1.

For each rhizobiome study, a co-occurrence network was constructed to provide insights into the structure and putative ecological interactions between taxonomic and functional core ASVs. Co-occurrence network analysis of the core rhizobiome revealed significant associations (with MIC values ranging from 0.22 to 0.91) between taxonomic and functional core ASVs for rice (564 significant associations), wheat (421 significant associations), maize (325 significant associations), citrus #1 (412 significant associations), citrus #2 (368 significant associations), sugarcane (268 significant associations), and tomato (325 significant associations), of which most associations (ranging from 82.5 to 86.8%) were non-linear (Fig. 4). Hub taxa belonging to both the taxonomic and functional core rhizobiomes were identified for each rhizobiome network, although hub taxa were mostly assigned (on average > 70%) to the functional core rhizobiome (Fig. 4; Table S6). These hub taxa were assigned to different genera depending on the core rhizobiome study (Fig. 4; Table S6).

## Discussion

Currently, the core rhizobiome of crops has been predominately defined on the basis of the taxonomic composition of the microbial community. However, this approach may only provide a partial characterization of the core rhizobiome as bacterial diversity in the rhizosphere is influenced by soil types^41^, plant genotypes^42–44^, and abiotic and environmental conditions^19,45,46^, thus potentially providing a different community composition for rhizosphere recruitment for crops of the same genotype. Indeed, taxa recruited by plants in the rhizosphere appear to have greater functional rather than taxonomic similarity^39^. Our results illustrate that a combined analysis of the taxonomic and functional description of the core rhizobiome rather than the use of a single taxonomic or functional approach provides a more complete characterization of agricultural crop core rhizobiomes and helps identify potentially important microbial drivers.

We performed an exploratory analysis of the differences in the taxonomic and functional core rhizobiomes using sequences from seven rhizobiome studies of six different crops (Table 1). Samples were analyzed using two different approaches (Fig. S1) in order to identify and characterize the taxonomic and functional core rhizobiome, and to detect possible crop-specific differences. For all rhizobiome studies examined, we found significant differences in the taxonomic (Fig. 1) and functional (Table 2) composition between the core rhizobiomes (Fig. 1), and different phyla (Fig. 2), genera (Fig. S3; Supplementary Data 1), pathways (Fig. 3), and KOs (Supplementary Data 2 and 3) dominated depending on the core rhizobiome type. This illustrates that using only a taxonomic or a functional approach could significantly influence conclusions about the core rhizobiome. For example, pathways and KOs assigned to traits related to plant hormone signal transduction and chemical structure transformations, as well as traits related to metabolic pathways, such as the metabolism of carbohydrates, amino acids, terpenoids, and polyketides were significantly more abundant (Supplementary Data 2) or exclusively present (Supplementary Data 3) in functional rather than taxonomic core rhizobiomes. By contrast, the taxonomic core rhizobiomes were significantly enriched in predicted pathways and KOs involved in genetic information processing, signal transduction, quorum sensing, biofilm formation, and membrane transport (Fig. 3; Supplementary Data 2 and 3). All of these functions are important for plant health (e.g., those involved in plant hormone balance, plant nutrition and disease suppression)^22,26,31^, however different taxa are potentially responsible for these functions leading to a different characterization of the core rhizobiome. For example, taxa of the phylum Proteobacteria dominated in both taxonomic and functional core rhizobiomes, while taxa from the phyla Acidobacteria, Bacteroidetes, Firmicutes, Nitrospirae, and Gemmatimonadetes had significantly different relative abundances in taxonomic and functional core rhizobiomes (Fig. 2).

**Table 1:** Summary of the sequence data used in this study, including the accession number, sequence type, region of the 16S rRNA gene, total number of samples, and total number of raw sequence reads for each rhizobiome study.

Crop	Accession number	Sequence type	Region of the 16S rRNA gene	Primer pairs	Total n. of samples	Total n. of raw sequence reads	Reference
Rice	SRP044745	Illumina Miseq	V4	515F and 806R	132	5,860,040	Edwards et al.^30^
Wheat	PRJNA209386	454 sequencing	V5–V7	799F and 1193R	44	66,345	Donn et al.^34^
Maize	PRJEB21985	Illumina Miseq	V4	515F and 806R	4866	627,638,736	Walters et al.^24^
Citrus #1	PRJNA362455	Illumina Miseq	V4	515F and 806R	23	640,251	Xu et al.^23^
Citrus #2	PRJNA636132, PRJNA636781	Illumina Miseq	V4	515Fa and 926R	624	11,245,456	This paper
Sugarcane	PRJNA390435	Illumina Miseq	V3–V4	341F and 805R	99	130,858,200	Hamonts et al.^32^
Tomato	PRJNA316593	Illumina Hiseq	V3–V4	341F and 805R	54	1,503,861	Cheng et al.^33^

**Table 2:** Variations in the functional composition of the rhizobiome between the taxonomic and functional core rhizobiomes at the pathway and KO levels as explained by weighted Bray–Curtis dissimilarity using the Adonis test.

Crop	Pathways	KOs
Rice	0.114***	0.144***
Wheat	0.187***	0.161***
Maize	0.241***	0.256***
Citrus #1	0.258***	0.321***
Citrus #2	0.245***	0.184***
Sugarcane	0.264***	0.244***
Tomato	0.287***	0.233***

R^2^ values are shown; 999 permutations.

*p < 0.05, **p < 0.01, ***p < 0.001.

In addition, for each rhizobiome study, there was a subset of genera that were exclusively present in one type of core rhizobiome (Supplementary Data 1). For example, genera such as *Syntrophorhabdus*, *Syntrophus,* and *Smithella* were only detected in the functional core rhizobiome of rice. Members from those genera are known for syntrophic growth with archaea and play a crucial role in methane production^54^. Thus, if only the taxonomic core was examined, these taxa would not be identified, leading researchers to miss potentially critical taxa in methane production, an important source of greenhouse gases from rice paddy soils^55^. Also, genera such as *Acinetobacter*, *Micromonospora*, *Phyllobacterium,* and *Serratia* were only detected in the functional core rhizobiome of both citrus #1 and citrus #2. Some species in these genera have been identified as plant growth promoting bacteria as they are able to fix atmospheric nitrogen, solubilize phosphate, produce siderophores and phytohormones, and enhanced salinity tolerance^56–58^. Thus, the use of only a taxonomic or functional approach may not only provide a partial characterization of core rhizobiome but also a different understanding of the processes leading to the recruitment of taxa in the core rhizobiome and the role of taxonomic and functional core taxa in nutrient cycling and plant growth. This supports the proposal of Lemanceau et al.^39^ for the implementation of a combined analysis of the taxonomic and functional description of the core rhizobiome to provide a more complete characterization.

The network analysis also illustrated that a combined analysis of the taxonomic and functional description of the core rhizobiome could help identify a greater number of potentially important taxa in the core rhizobiome (Fig. 4; Table S6). For all rhizobiome studies, highly interconnected taxa (defined here as “hub” ASVs) were identified as belonging to both taxonomic and functional core rhizobiomes but were associated with different genera based on the type of core rhizobiome (Table S6). Thus, by looking at only hub taxa in the taxonomic or functional approach we may not identify all the potentially important taxa in the core rhizobiome which could subsequently affect the design of future agricultural microbiome experiments. Multiple microbes affiliated with the hub taxa detected in the taxonomic and functional core rhizobiomes (e.g. those of the genera *Azospirillum*, *Azotobacter, Bradyrhizobium, Burkholderia*, *Nitrospira, Nitrosomonas*, *Pseudomonas, Rhizobium,* and *Streptomyces*) have been identified as plant beneficial microbes, and might help maintain plant hormone balance, control root development, facilitate nutrition acquisition, and prevent disease in the plant host^39,59^. Future research is needed to experimentally verify the role of those potentially important taxa to a specific plant’s health. In addition, numerous significant and non-linear associations between ASVs belonging to taxonomic and functional core rhizobiomes were detected in the microbial network analysis. In the rhizosphere, interactions between microbial species are often non-linear and can be either regulated by factors within the microbial community itself or by environmental factors^32,47,59,60^. To what extent the recruitment of particular microbial groups in the core rhizobiome is controlled by the plants or is also indirectly affected by abiotic environmental and soil physico-chemical factors remains to be investigated.

The use of a taxonomic or functional approach also provided different insights into functional redundancy in the core rhizobiome. We detected a significantly greater number of ASVs (Table S2), pathways, and KOs (Fig. 2; Table S5; Supplementary Data 2) in functional rather than taxonomic core rhizobiomes, and functional core ASVs were also affiliated with different phyla (Fig. 2) and genera (Fig. S3). These results illustrate less functional redundancy in the functional rather than taxonomic core rhizobiomes. This is not unexpected, as the recruitment of microbial populations belonging to different taxa but sharing the ability to ensure key functions for the host plant has been identified in *Arabidopsis*^43^, barley^22^, cumbuber^26^, and *Jacobea vulgaris*^47^. In addition, pathways and KOs in the functional core rhizobiomes were mainly assigned to central metabolism functions of bacteria. These functions are probably not specific to the rhizosphere and are likely present in other microbiomes (e.g., aquatic sediments, animal, and water treatment plants).

We detected similar core microbiome members and predicted functions among phylogenetically related plants such as maize and sugarcane, citrus #1 and citrus #2, and to a lesser extent rice, wheat, and tomato (Figs. 2 and 3; Supplementary Data 2^61^). These similarities not only support the frequently reported theory that plant genotypes recruit microbial functions to complement their own functions (e.g.^39,62,63^), but also offer exciting opportunities to determine if there is a common host-independent core microbiome. To begin this exploration, we provide a list of potentially important microbes of the core rhizobiome (Table S5) for investigation into specific plant-microbiome interactions of crops. This information is required to isolate and inoculate plants with selected microbes^5^. For example, these microorganisms could be automatically inoculated to plants using the RootChip^64^ or layered microfluidic devices^65^, which place sterilized seeds/seedlings on the culture/co-culture media of selected core microbes. The use of high-coverage sequencing techniques should also be prioritized in the future to identify these potentially important taxa at the species taxonomic level and to try to isolate them using cultivation-based efforts.

There are currently two primary methods available to determine microbial community functions: (1) software such as PICRUSt2 or Tax4Fun^66^ that predict microbial community functions from taxonomic profiles (amplicon sequences), since microbial functional profiles cannot be directly identified using 16S rRNA gene sequence data owing to strain variation; and (2) shotgun metagenome sequencing which provides information on the relative abundance of the taxonomic composition as well as functional genes. Although the utility of PICRUSt2 for inference of predicted functions was validated using both amplicon and metagenome sequencing^37^, recent studies have shown that the performance of PICRUSt2 may be limited outside of human samples^67,68^. However, the tools remain useful for predicting microbial functions of environmental studies and providing initial assessments of the core rhizobiome, as has been shown in examination of core rhizobiomes^28^, rhizosphere soil (e.g.^69^), water (e.g.^70^), fish (e.g.^71^), and human gut (e.g.^72^). Use of these software tools is particularly helpful for initial crop core rhizobiome studies because the current cost of metagenome sequencing hinders its application, as a large number of samples are necessary in order to ensure adequate statistical power for detecting true differences^73^. Additionally, metagenome sequencing can be very challenging for low biomass samples or samples that are dominated by non-microbial DNA^74^. Currently, the use of shotgun metagenome sequencing to characterize core rhizobiomes is limited^23,26,31^, but in the future it could likely provide more reliable identification of the microbial community composition and functions in the core rhizobiome, and may further refine our findings as functions were predicted from taxonomy in our study.

In summary, our results illustrate that a combined analysis of the taxonomic and functional description of the core rhizobiome rather than the use of a single taxonomic or functional approach provides a more complete characterization of agricultural crop core rhizobiomes, as previously suggested^14,39^. A better characterization of core microbiomes and their potentially important microbial drivers may provide increased understanding of host-microbe interactions occurring not only in the rhizosphere but also in other ecological subjects such as animal ecology^20^. In addition, the identification of taxonomic and functional core rhizobiomes might be important for understanding the potential impacts of agricultural practices (e.g., conventional vs. organic), soil types, and abiotic factors on plant–microbe interactions of high-value crops^35^, which could be utilized to understand soil quality^75,76^ and improve agriculture sustainability. So far, the characterization of core rhizobiomes of crops have been mainly focused on bacterial communities. Few studies have looked at the potential role of fungi^23,32^, archaea^30^, protist, and viruses in the core rhizobiome. A better understanding of the relationships between archaeal, bacterial, and fungal communities in the core rhizobiome of crops may provide additional insights into the host-microbiome interactions that contribute to increased plant nutrient uptake and resistance to biotic and abiotic stresses^77^ and help to optimize and maximize future agricultural microbiome engineering solutions^5^.

## Methods

### Sequencing data

Raw sequencing reads of the 16S rRNA gene from previously published rhizobiome studies of rice^30^ (*Oryza sativa*), wheat^34^ (*Triticum aestivum*), maize^24^ (*Zea mays*), citrus^23^ (*Citrus sinensis*) (herein citrus #1), surgarcane^32^ (*Saccharum officinarum*), and tomato^33^ (*Solanum lycopersicum*) were obtained from the National Center for Biotechnology Information (NCBI) Sequence Read Archive (SRA) database (Table 1). Details of planting, handling, and collection of the rhizosphere samples can be found in the original references. In addition, we included a subset of 624 samples from the rhizosphere of citrus (*C. sinensis; C. paradise; C. reticula;* and *C. grandis*) in Florida (herein citrus #2; Table 1), whose information on sample collection, DNA extraction, and sequencing is available in the Supporting Material (Methods S1; Table S1).

### Sequencing analysis

Raw reads from the 16S rRNA gene obtained from each rhizobiome study were analyzed individually due to different primer pairs (Table 1). All bioinformatic analyses were performed study by study. Raw sequence reads were analyzed using QIIME2 v2018.4^78^. Reads were trimmed where the average quality score dropped below 25, and dereplicated using DADA2^79^ with paired-end setting, resulting in ASV tables containing read counts. ASVs were assigned to the SILVA 132 database^80^, using naïve Bayes classifier in QIIME2^81^, which produced taxonomy tables. ASVs matching “Chloroplast” and “Mitochondria” were removed from the data set. Good´s coverage index for each sequencing sample was estimated as 1 – (S/N), where S is the number of unique ASVs and N the number of observed ASVs. A total of 777,812,889 raw sequence reads were obtained from the 5842 samples used in this study (Table 1), of which 330,180,919 high-quality sequences were retained after the sequencing analysis (Table S2).

### Identification of the taxonomic core rhizobiome and its potential functional traits

A flow-chart describing how the taxonomic core rhizobiomes were identified and characterized is shown in Fig. S1A. For each rhizobiome study, ASVs present in at least 75% of the samples were identified using the QIIME2 feature-table core-features command and named “taxonomic core ASVs.” To better understand the potential functions of the detected taxonomic core ASVs, each ASV table was filtered using the QIIME2 feature-table filter-features and feature-table filter-samples commands to specifically select for the taxonomic core ASVs present in each sample. Filtered samples were renamed “taxonomic core rhizobiomes.” For each rhizobiome study, the functional potential of the taxonomic core rhizobiome containing the taxonomic core ASVs was determined using the PICRUSt2 script pathway_pipeline.py at both the pathway and KO levels as previously described by Douglas et al.^37^. Taxonomic core ASVs assigned to different KOs were identified in this analysis to account for functional redundancy^48^. To date, methods to determine microbial community functions are primarily based on tools such as PICRUSt2^37^ or Tax4Fun^51^ that predict functional profiles from 16S rRNA gene sequences. Shotgun metagenome sequencing can also be used to characterize the functions of a microbial community but its high cost and low resolution when there is low microbial biomass limits its use^52^. While the accuracy of these prediction tools appears to vary across samples types and functional categories^52^, they can still provide a method to explore potential functional differences in microbial communities.

### Identification of the functional core rhizobiome and its potential functional traits

A flow-chart describing how the functional core rhizobiomes were identified and characterized is shown in Fig. S1B. For each rhizobiome study, the functional potential of the samples was first determined using the PICRUSt2 pathway_pipeline.py script^37^. The analysis created a ko_metagenome.qza output file which contained the KO rhizobiome predictions (rows are KOs and columns are samples). KOs present in at least 75% of the samples were identified as “core KOs.” We then selected ASVs assigned to core KOs in at least 75% of the samples using PICRUSt2 and the QIIME2 feature-table core-features command and named those ASVs as “functional core ASVs.” ASVs assigned to more than one core KO were also included in this analysis to account for functional redundancy^48^. Each ASV table was then filtered using the QIIME2 feature-table filter-features and feature-table filter-samples commands to specifically select for the functional core ASVs present in each sequencing sample. Filtered samples were renamed “functional core rhizobiomes.” To look at the overlap of functional traits between the taxonomic and functional core rhizobiomes, for each rhizobiome study the functional potential of the functional core rhizobiome containing the functional core ASVs was determined using the PICRUSt2 pathway_pipeline.py script at both the pathway and KO levels^37^.

While the cut-off value of 75% has been previously used to select for the taxonomic or functional core rhizobiome of at least three crops (e.g.^23,28,32^), cut-off values of 85%^33^, 95%^24^, and 100%^25^ have also been used. As there is not a consensus on cut-offs to characterize core rhizobiomes on the basis of taxonomic and/or functional traits^18,20^, we first assayed the effect of cut-offs ranging from 70 to 90% on the taxonomic and functional composition of the core rhizobiome. Regardless of the rhizobiome study, significant differences in the taxonomic and functional composition for each taxonomic and functional core rhizobiome were not observed with cut-offs of 75% or above for all rhizobiome studies (data not shown). Thus, cut-offs of 75% were used in this study (Fig. S1).

### Statistical analyses

For each rhizobiome study, the bacterial community composition of the taxonomic and functional core rhizobiomes were ordinated by principal coordinates analysis (PCoA) on Bray–Curtis and unweighted UniFrac distance matrices^82^ using the pcoa() function from the R package Ape^83^. Sequencing samples were not rarified in our study as suggested by McMurdie and Holmes^84^, but clustering in the PCoA was not due to different number of sequences between samples (data not shown). Differences in bacterial community composition between core rhizobiomes were tested by analysis of similarities (ANOSIM). Variations in the functional composition of the rhizobiome between the taxonomic and functional core rhizobiomes at the pathway and KO levels were assayed using the Adonis test (999 permutations) for each rhizobiome study^85^. To do this, the output files “ko_metagenome.qza” and “pathway_abundance.qza” of the PICRUSt2 analysis were transformed to Biological Observation Matrix (BIOM) tables and used for the generation of beta-diversity (weighted Bray–Curtis dissimilarity) metrics. Significant differences in the number and relative abundance of ASVs at the phylum and genus levels, and predicted functions at the pathways and KOs levels between the taxonomic and functional core rhizobiomes were calculated using the Welch’s t-test^86^ and the Benjamini–Hochberg False Discovery Rate (FDR) multiple-test correction^87^ using the R package ‘sgof’. Phyla, genera, pathways, and KOs showing significant differences between taxonomic and functional core rhizobiomes (*p*-values < 0.05) were retained^69,88^. The Benjamini–Hochberg FDR multiple-test correction was used to avoid type 1 and 2 errors. Sequences of the ASVs identified in the taxonomic and functional core rhizobiomes were used for phylogenetic analyses. Distances were calculated according to Kimura’s two-parameter model^89^ and phylogenetic trees were inferred using maximum-likelihood (ML) and the MEGA 7.0^90^ software.

### Co-occurrence network construction and analysis

For each rhizobiome study, a co-occurrence network was constructed to provide insights into the structure and putative ecological interactions between taxonomic and functional core ASVs belonging to taxonomic and functional core rhizobiomes, and to study whether hub taxa can belong to one or both types of core rhizobiomes. BIOM tables containing taxonomic and functional core ASVs served as an input matrix for the co-occurrence network analysis. We first used the Habitat Filtering (HF) correction algorithm as implemented in the HabitatCorrectedNetwork.py script^91^ as some of the samples included in this analysis were collected from different fields/locations. The HF is a novel correction algorithm that reduces habitat effects (e.g., sample site), and has been used to construct co-occurrence networks from microbial sequencing data^91,92^. Second, the maximal information-based non-parametric exploration analysis^32,90^ was performed to detect significant associations between taxonomic and functional core ASVs. For each rhizobiome study, the maximal information coefficient (MIC^90^) was measured to determine the dependence of ASVs in the core rhizobiome using the pair’s relative abundance in all samples. Significant MIC scores (adjusted *p* < 0.05) were determined by the Benjamini and Hochberg procedure using a false discovery rate of 5% as previously described^32^. Significant associations were visualized using Cytoscape v3.7.1^93^. Highly interconnected ASVs (i.e., potential hub taxa^49,50^) were identified within each microbial network as the ASVs with the highest values for degree (number of neighbours/correlations in the network), closeness centrality (1/[(distance to all other nodes]), and betweenness centrality (fraction of shortest paths the node is on)^32,48,94,95^, as calculated in Cytoscape v3.7.1^93^.

## Supplementary Information

Supplementary Information 1.

Supplementary Data 1.

Supplementary Data 2.

Supplementary Data 3.

Supplementary Materials.
