# Supplementary material for: Insights into the taxonomic and functional characterization of agricultural crop core rhizobiomes and their potential microbial drivers
Source: Sci Rep. 2021 May 12;11:10068. doi: 10.1038/s41598-021-89569-7 (PMC8115259; doi:10.1038/s41598-021-89569-7)
Supplement: Supplementary file 1 — Supplementary Information 1. [file 41598_2021_89569_MOESM1_ESM.docx]

**Additional Supplementary Files:**

**Title:** Insights into the taxonomic and functional characterization of agricultural crop core rhizobiomes and their potential microbial drivers

**Authors:** Antonio Castellano-Hinojosa and Sarah L Strauss^*^

**Affiliation**: University of Florida Southwest Florida Research and Education Center, 2685 State Rd 29N, Immokalee, FL, USA 34142, 239-658-3468

***Corresponding author:**

Sarah L. Strauss

University of Florida Southwest Florida Research and Education Center, 2685 State Rd 29N, Immokalee, FL, USA. 34142. Tel: +1 239-658-3468; E-mail: strauss@ufl.edu

**Supplementary Data 1.** List of genera that were exclusively detected in either the taxonomic or functional core rhizobiomes. An individual sheet per rhizobiome study (rice, wheat, maize, citrus #1, citrus #2, sugarcane, and tomato) is included.

**Supplementary Data 2.** List of KOs whose relative abundances were significantly different between taxonomic and functional core rhizobiomes according to the Welch’s t-test and Benjamini–Hochberg FDR multiple test correction. The type of core rhizobiome in which the KO had a significantly higher relative abundance is indicated. *p*-values < 0.05 were considered significant. An individual sheet per rhizobiome study (rice, wheat, maize, citrus #1, citrus #2, sugarcane, and tomato) is included.

**Supplementary Data 3.** List of KOs that were exclusively detected in either the taxonomic or functional core rhizobiomes. An individual sheet per rhizobiome study (rice, wheat, maize, citrus #1, citrus #2, sugarcane, and tomato) is included.
