# Supplementary material for: Insights into the taxonomic and functional characterization of agricultural crop core rhizobiomes and their potential microbial drivers
Source: Sci Rep. 2021 May 12;11:10068. doi: 10.1038/s41598-021-89569-7 (PMC8115259; doi:10.1038/s41598-021-89569-7)
Supplement: Supplementary file 5 — Supplementary Materials. [file 41598_2021_89569_MOESM5_ESM.docx]

**Supplementary Material**

Insights into the taxonomic and functional characterization of agricultural crop core rhizobiomes and their potential microbial drivers

**Authors:** Antonio Castellano-Hinojosa and Sarah L Strauss^*^

**Affiliation**: University of Florida Southwest Florida Research and Education Center, 2685 State Rd 29N, Immokalee, FL, USA 34142, 239-658-3468

***Corresponding author:**

Sarah L. Strauss

University of Florida Southwest Florida Research and Education Center, 2685 State Rd 29N, Immokalee, FL, USA. 34142. Tel: +1 239-658-3468; E-mail: strauss@ufl.edu

**Methods S1**

Sample collection

Rhizosphere soil samples were collected from six citrus (*Citrus sinensis* [L.] Osbeck) rootstock genotypes in five citrus orchards in Florida, USA (Table S1). According to the Soil Taxonomy of USDA, the soil at the field sites is an Immokalee fine sand (arenic, alaquods, sandy, siliceous, hyperthermic), with a nearly flat slope (0–2%), low runoff class, and poor natural drainage. Regardless of the field site, all trees were affected by Huanglongbing (HLB) disease. Samples were collected from randomly-selected citrus rows. Rhizosphere samples were collected from 10 randomly-selected trees per row from the top 0-30 cm depth, 30 cm from the trunk, and pooled together. Rhizosphere soil was collected from the tree roots by placing the tree roots in 50-mL Falcon tubes containing 15-mL of phosphate-buffered saline (PBS). Tubes were shaken for 60 s and centrifuged at 6000 rpm for 1 min. The roots were removed, and the pelleted material considered the rhizosphere soil. Rhizosphere soil samples were stored at -80 °C until use.

DNA extraction and sequencing

DNA from 0.25 g of rhizosphere soil for each sample was extracted using the DNeasy PowerSoil PowerLyzer Kit (Qiagen, Germantown, MD, USA) following the manufacturer’s instructions. The extracted DNA was quantified using a Qubit Fluorometer (Thermofisher Scientific) through the Quant-iT dsDNA HS Assay Kit and was sent for normalization, library preparation, and sequencing at the DNA Services Facility at the University of Illinois, Chicago, IL, USA. The V4 region of the 16S rRNA genes was amplified using the 515Fa and 926R primers following the EMP protocol^1^.

**Functional traits of the**

**taxonomic core rhizobiome samples**

compute_core_microbiome.py script

PICRUSt2 script pathway_pipeline.py

filter_ASVs_from_ASV_table.py script

**A.**

**ASV 2**

**List of taxonomic core ASVs**

**ASV 1**

**ASV n**

**KOs/ pathways**

**Taxonomic core rhizobiome samples**

**Taxonomic core ASVs**

n ASVs

**>75% of the samples**

n samples

n samples

n samples

n samples

**Functional traits of the**

**functional core rhizobiome samples**

**B.**

PICRUSt2 script pathway_pipeline.py

filter_ASVs_from_ASV_table.py script

PICRUSt2 script pathway_pipeline.py

PICRUSt2 script pathway_pipeline.py

**KO 2**

**KO 1**

**KO n**

KOs/ pathways

**Functional core rhizobiome samples**

**Functional core ASVs**

**List of functional core ASVs**

**List of core KOs**

**Core KOs**

n samples

**>75% of the samples**

n ASVs

**>75% of the samples**

**Fig. S1.** A flow-chart describing how the taxonomic **(A)** and functional **(B)** core rhizobiomes were identified and characterized. Microsoft PowerPoint was used to create this figure.

n samples

n samples

n samples

**ASV n**

**ASV 1**

**ASV 2**

n samples


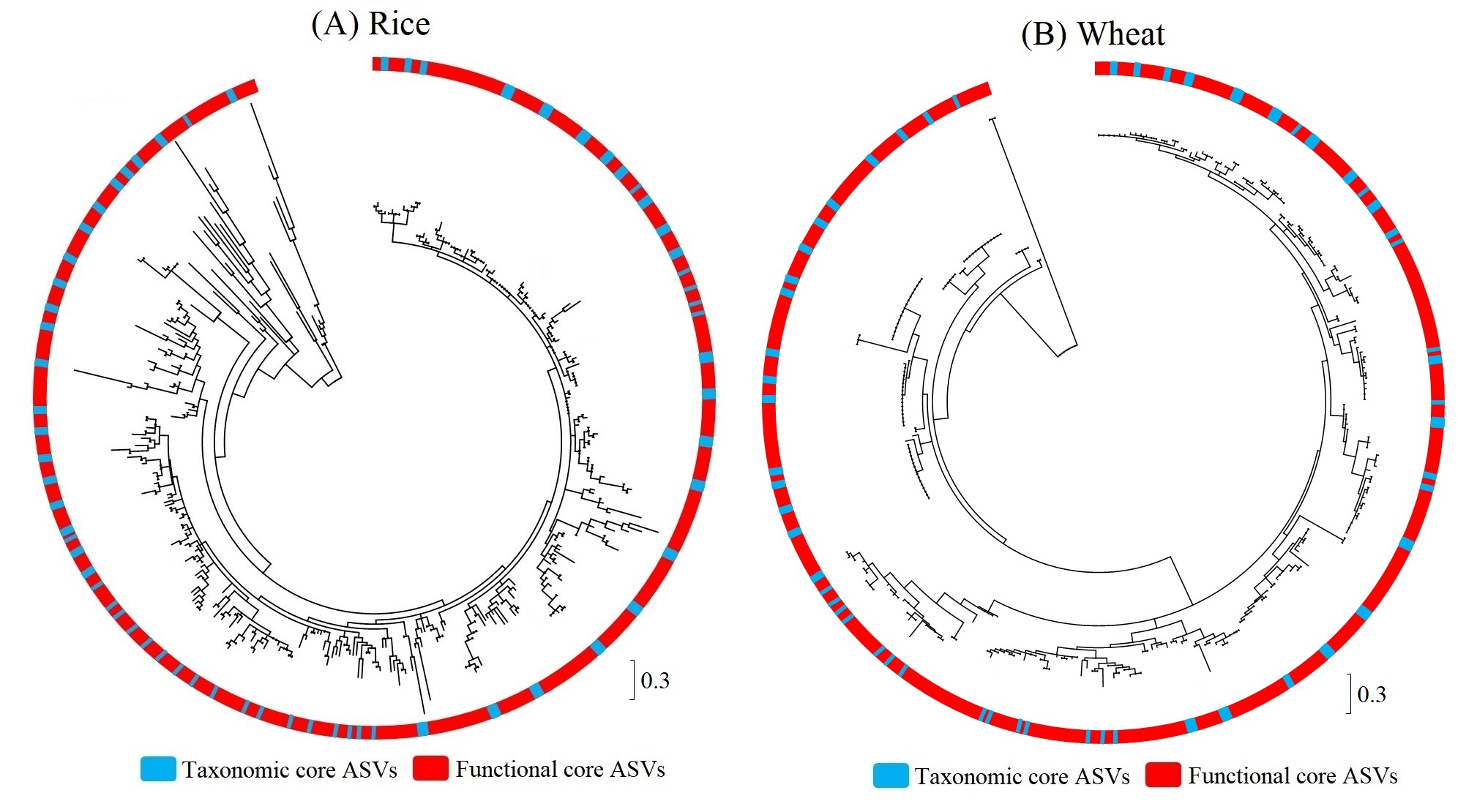


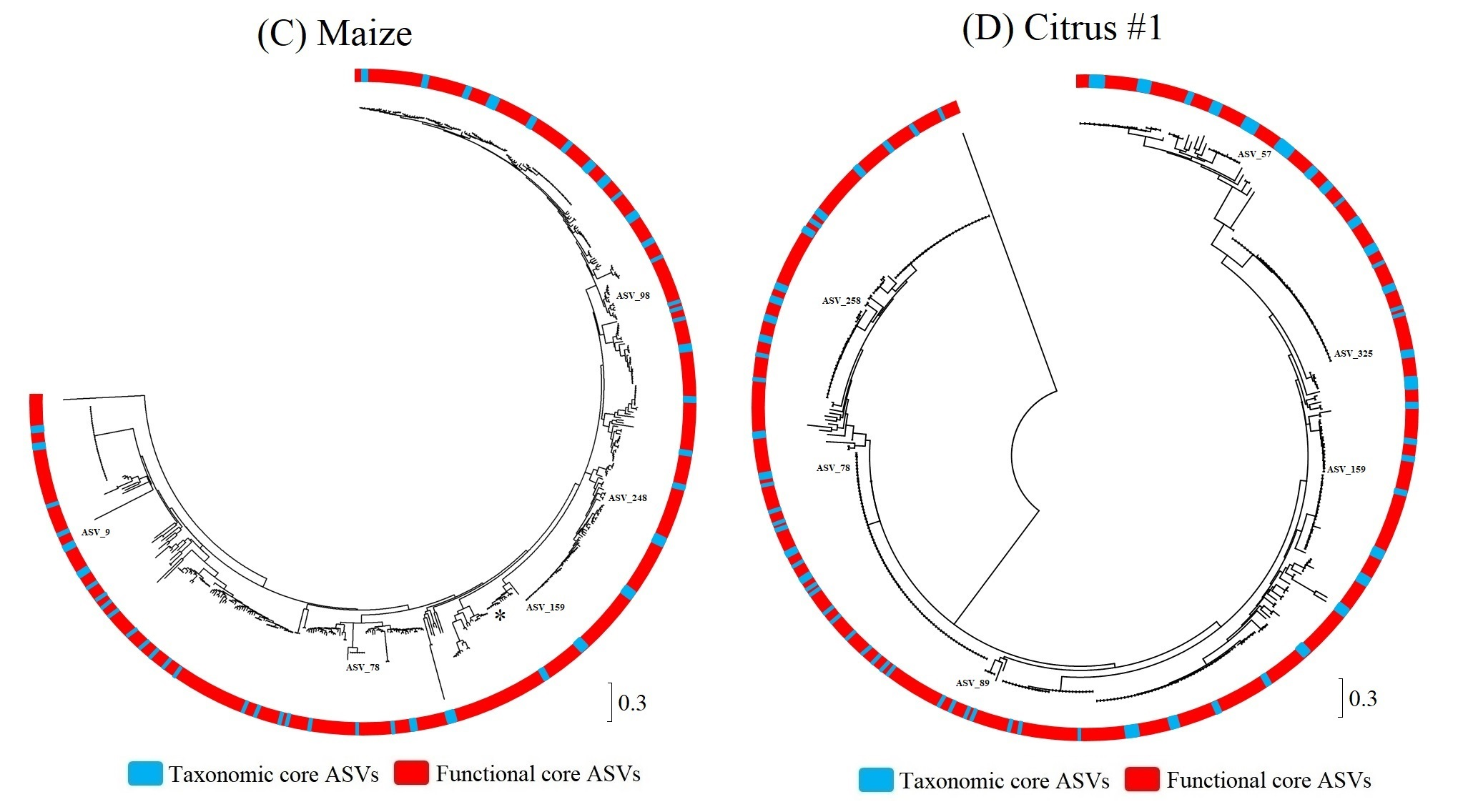


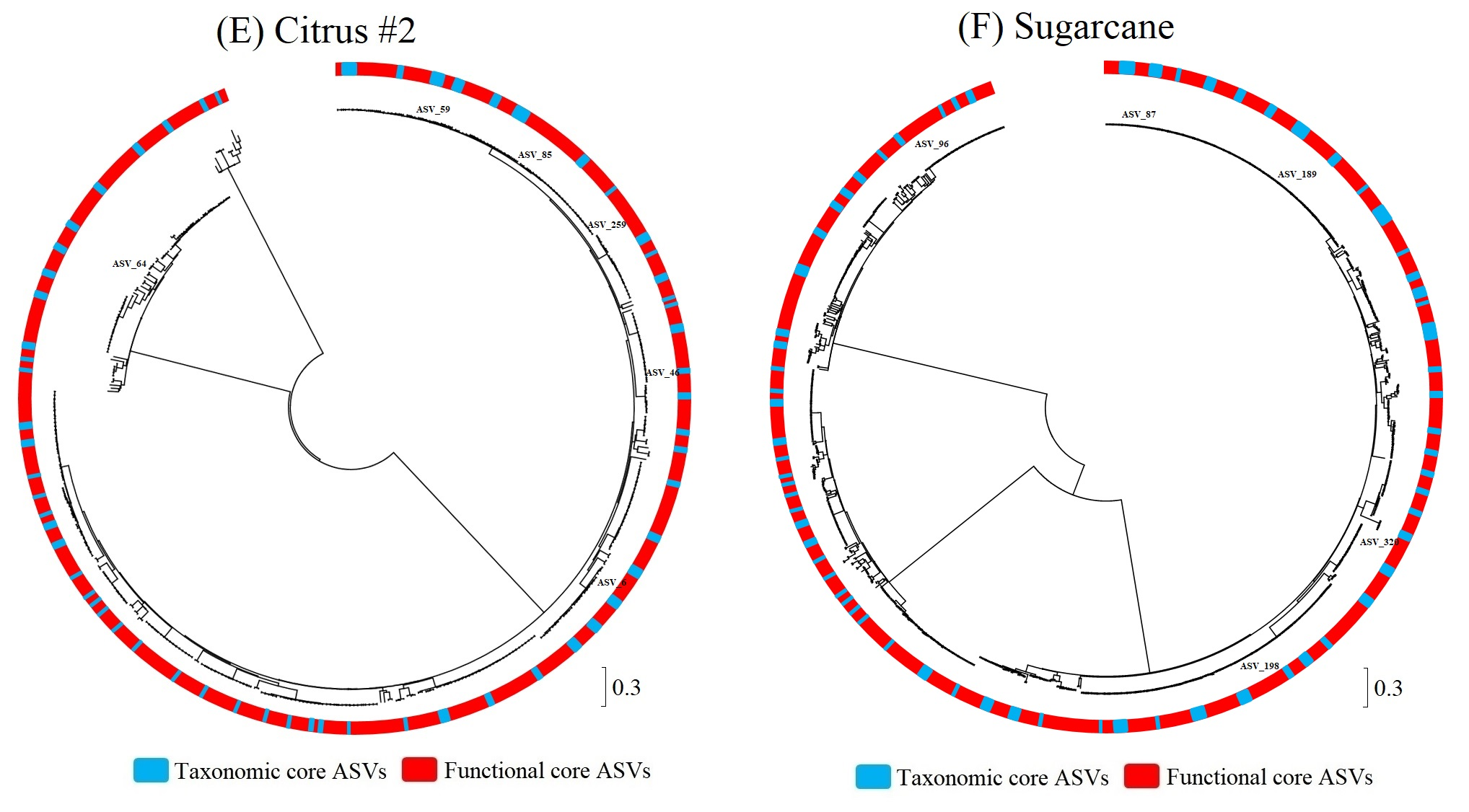


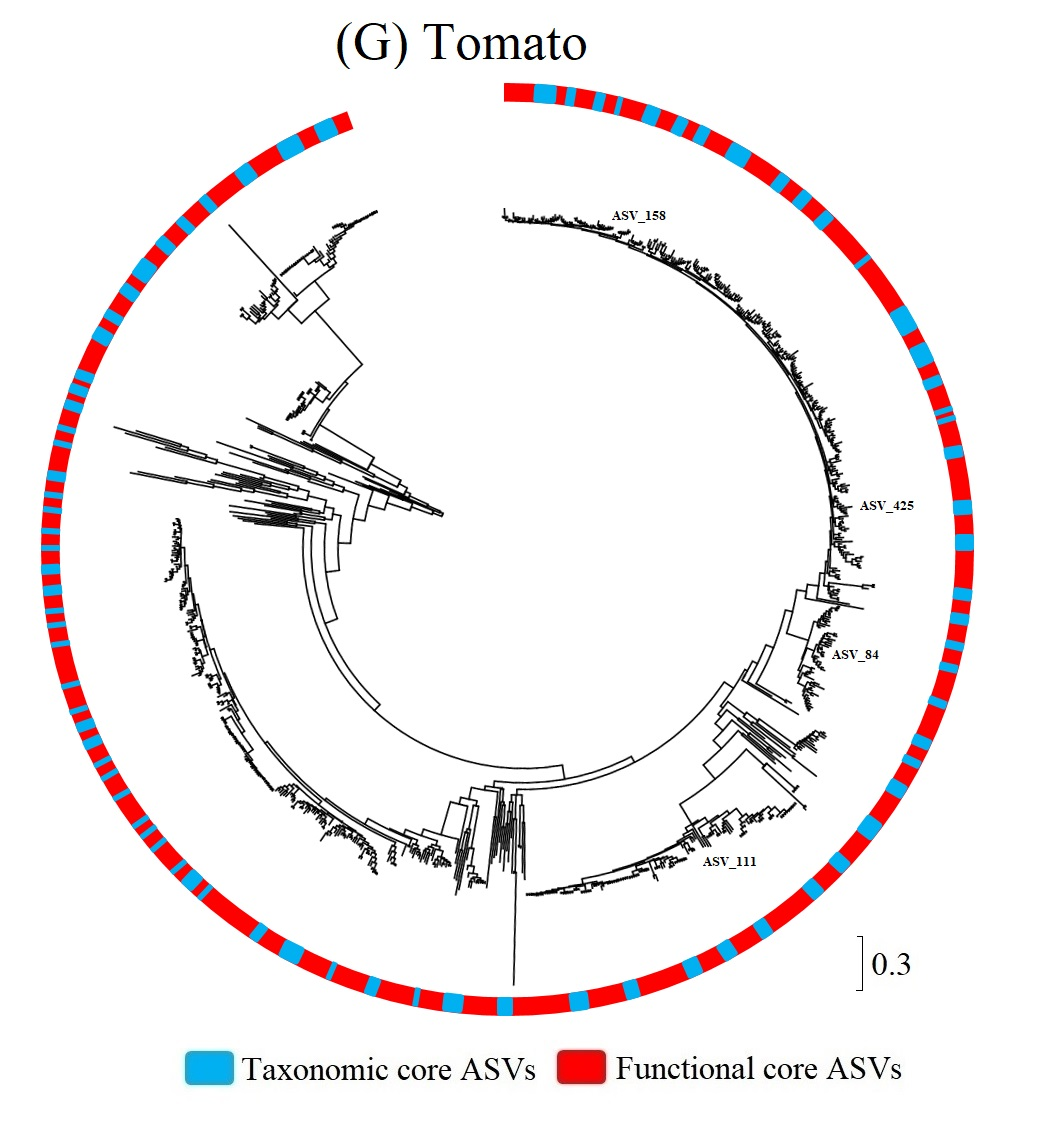


**Fig. S2.** Maximum likelihood phylogenetic tree of ASVs identified in the taxonomic and functional core rhizobiome for rice (A), wheat (B), maize (C), citrus #1 (D), citrus #2 (E), sugarcane (F), and tomato (G) (Table S3). Bar, 30 nucleotides substitution per 100 nucleotides derived from 1000 replications. ASVs identified as “hub taxa” in subsequent analyses are highlighted in the trees (Fig. 4; Table S6). Phylogenetic trees were created using Mega 7.0.

**
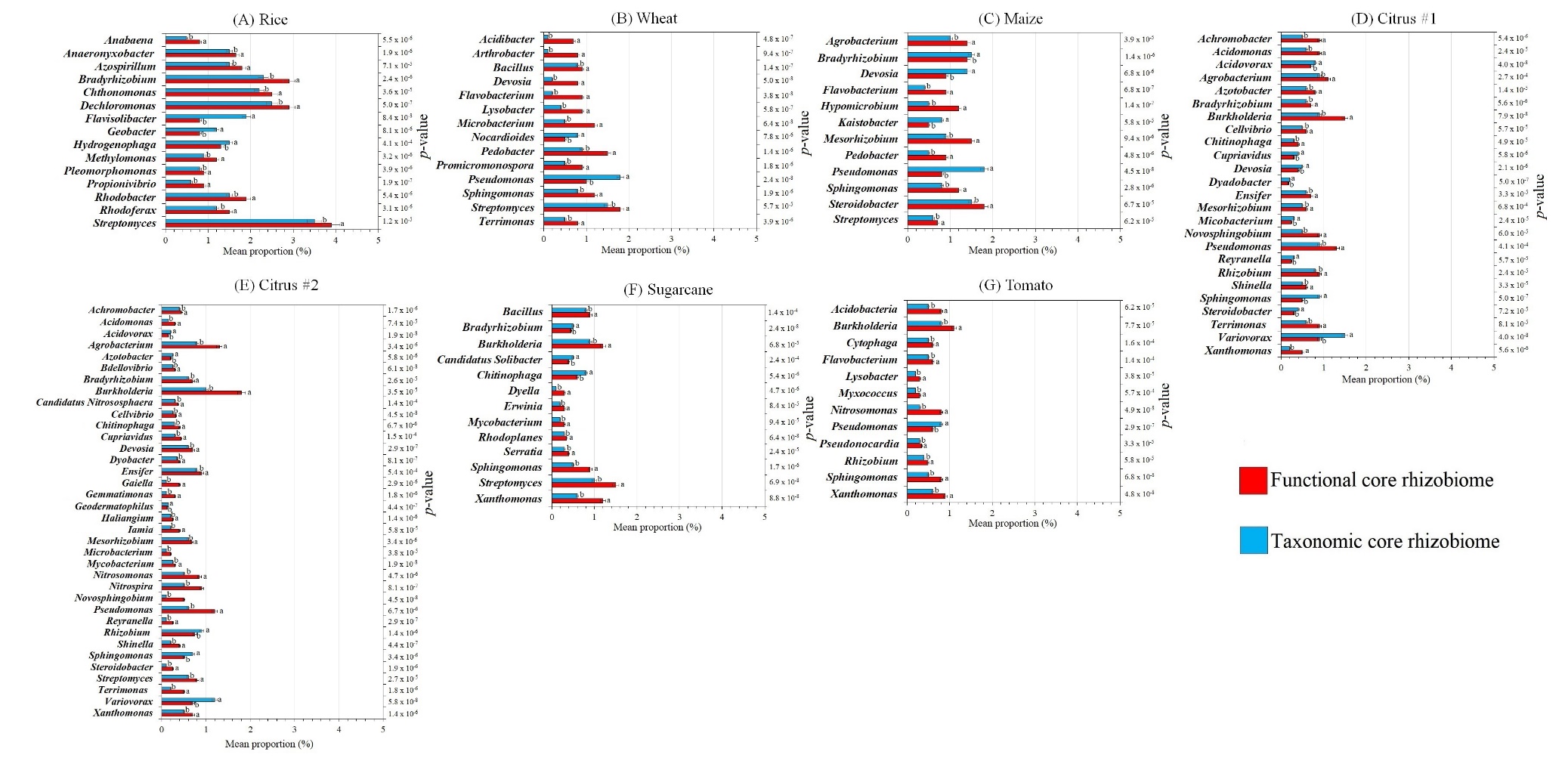
**

**Fig. S3.** Histograms of genera significantly different between taxonomic and functional core rhizobiomes according to the Welch’s t-test and Benjamini–Hochberg FDR multiple test correction for rice (A), wheat (B), maize (C), citrus #1 (D), citrus #2 (E), sugarcane (F), and tomato (G). Letters above the bars indicate significant differences in the relative abundance of each genus between taxonomic and functional core rhizobiomes*,* and *p*-values < 0.05 were considered significant. Bars marked with the same letter are not significantly different.

A.

A1 A2 A3


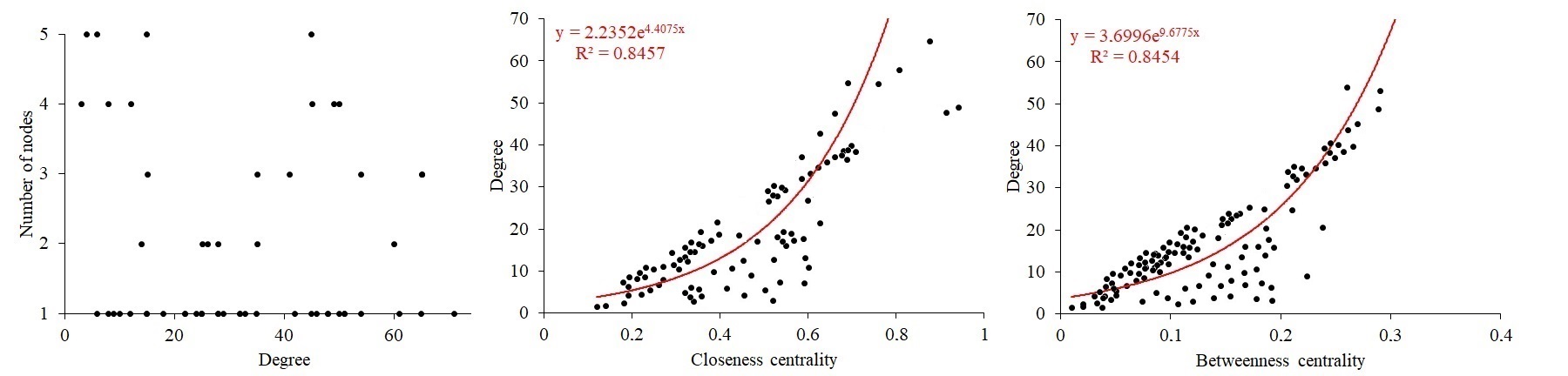


B.

B1 B2 B3


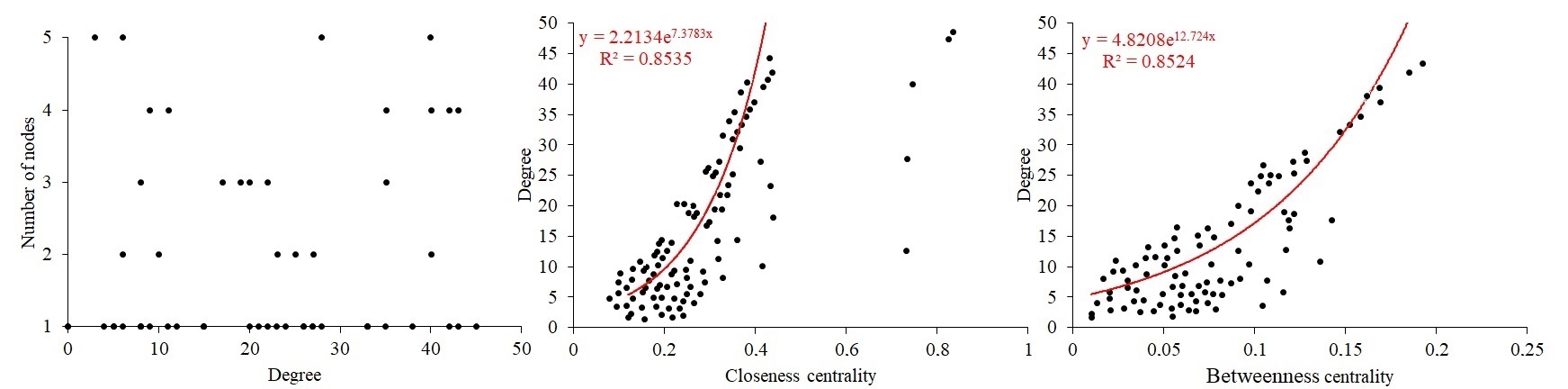


C.

C1 C2 C3

**
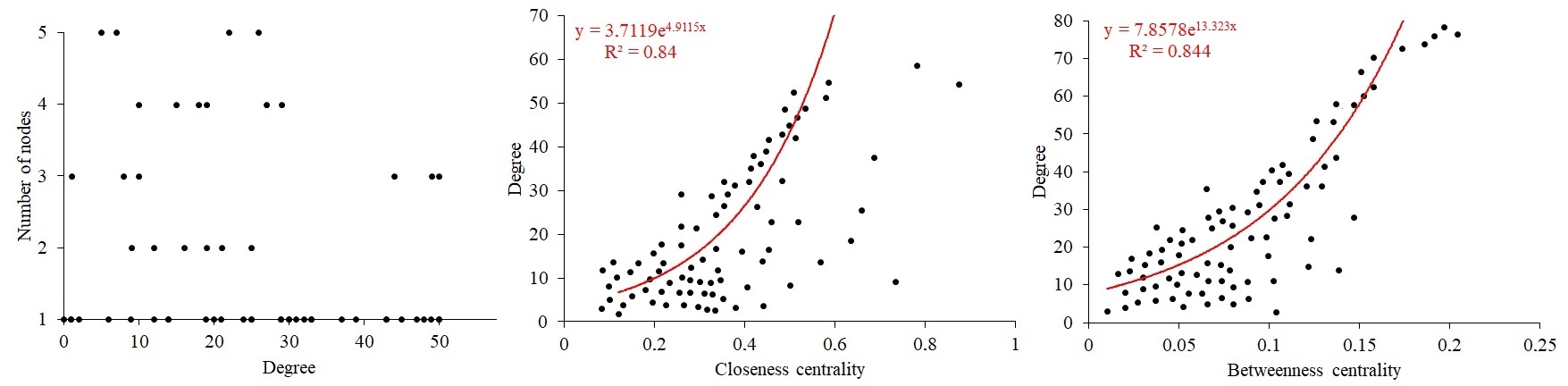
**

D.

D1 D2 D3

**
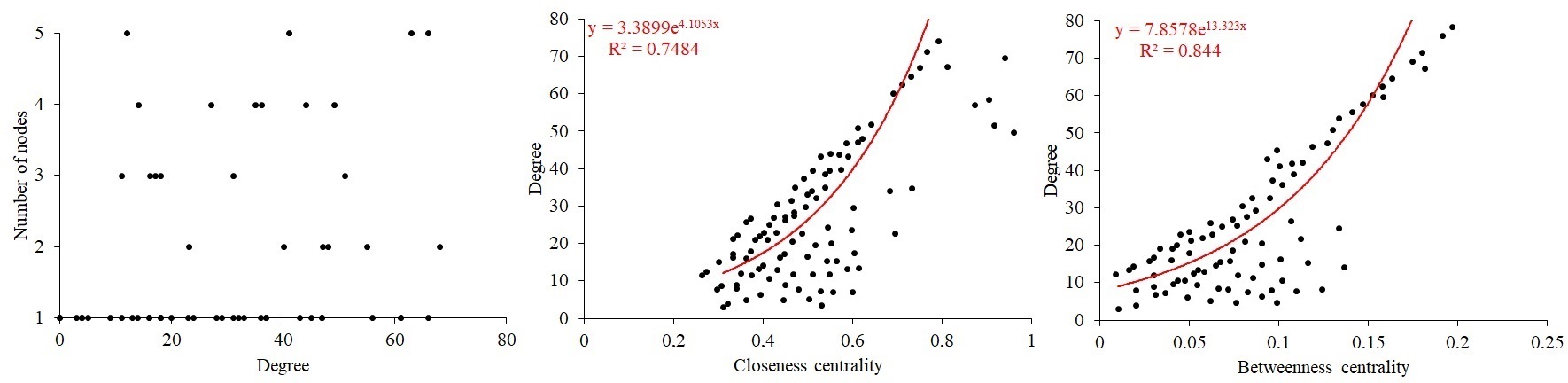
**

E.

E1 E2 E3

**
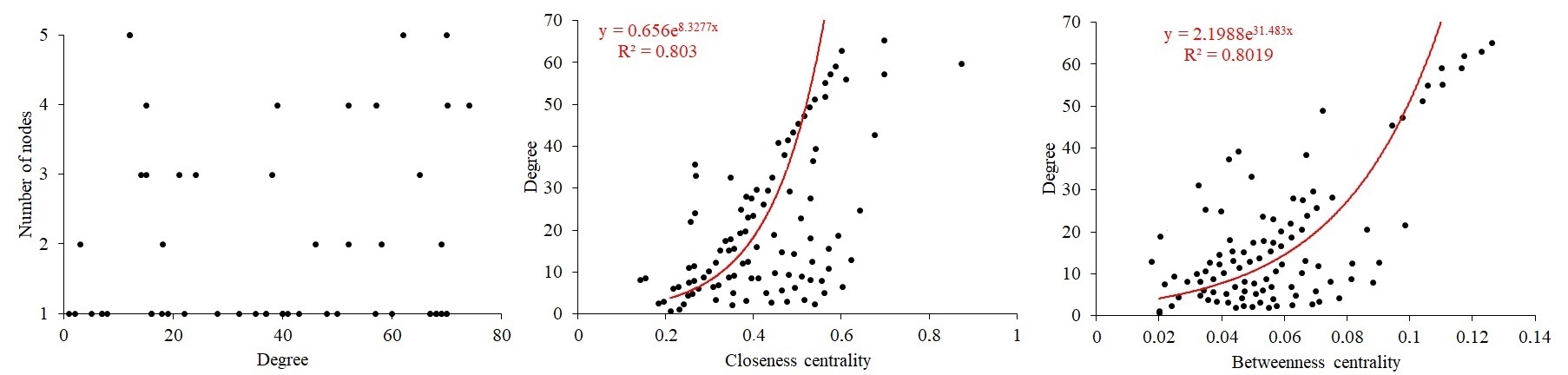
**

F.

F1 F2 F3

**
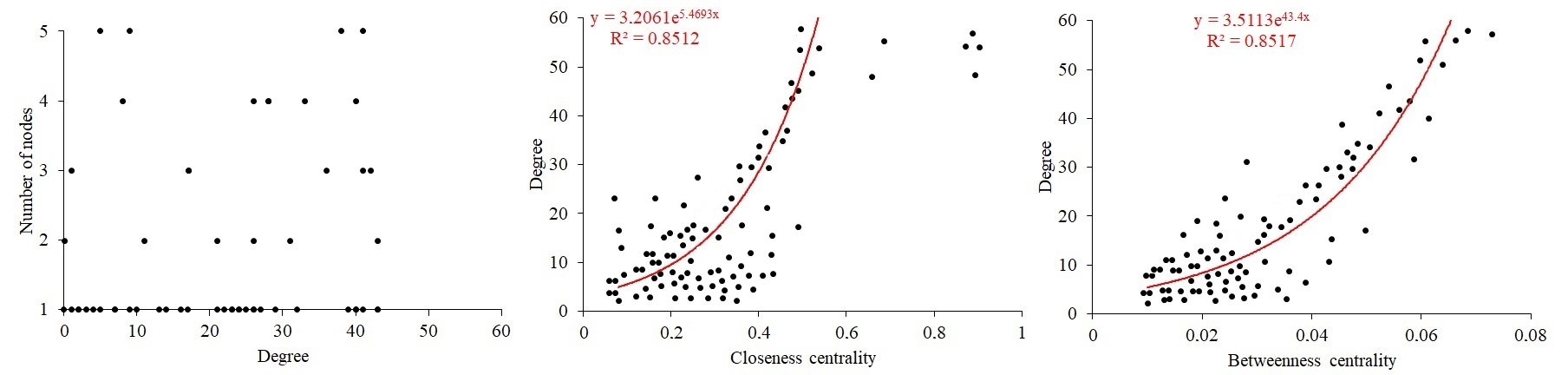
**

G.

G1 G2 G3

**
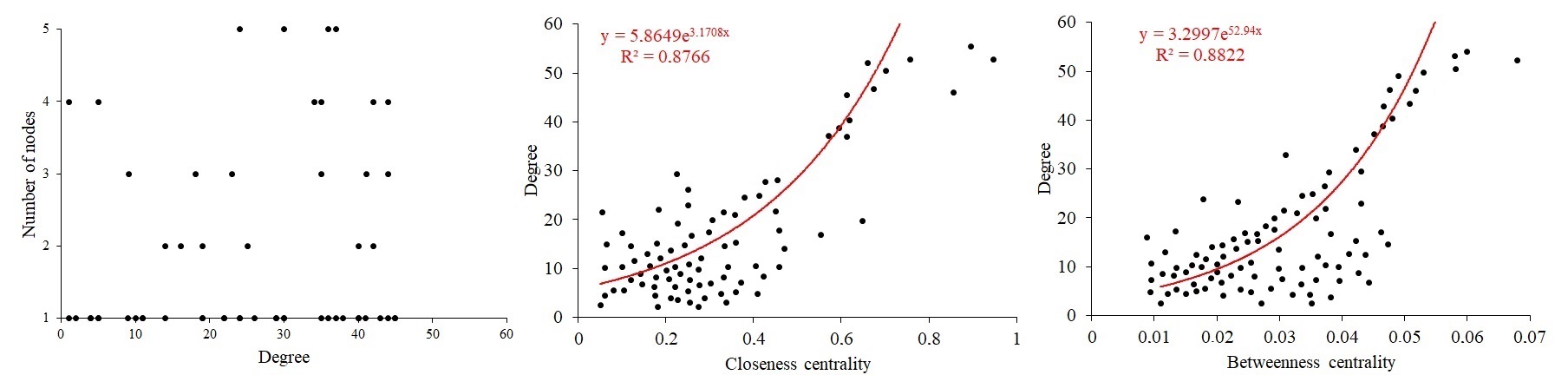
**

**Fig. S4.** Identification of hub taxa in the rice (A), wheat (B), maize (C), citrus #1 (D), citrus #2 (E), sugarcane (F), and tomato (G) rhizobiome networks displayed in Fig. 4. Hub taxa were selected as those taxonomic core ASVs or functional core ASVs that were more central in the network based on their degree (A1, B1, C1, D1, E1, F1, and G1), closeness centrality (A2, B2, C2, D2, E2, F2, and G2) and betweenness centrality (A3, B3, C3, D3, E3, F3, and G3). A power law curve fitted to plots (A2, B2, C2, D2, E2, F2, and G2) and (A3, B3, C3, D3, E3, F3, and G3) is shown in red. Cut-offs for degree, closeness centrality, and betweenness centrality were selected and indicated by red dashed lines in the plots as described by Hamonts et al.^2^. Hub taxa were selected as those ASVs that satisfied at least two of the three selected cut-offs and are listed in Table S6.

**Table S1.** Overview of the field site locations and number of samples collected from the rhizosphere of citrus trees (citrus #2) in Florida.

| County | Rootstock | Scion | Parentage/species | Tree age (years) | Sampling date | No. samples |
| --- | --- | --- | --- | --- | --- | --- |
| Collier | Swingle | Valencia | *C. paradisi* Macf. × *Poncitrus trifoliata* | 29 | August 2019 | 30 |
| Hendry | Swingle | Valencia | *C. paradisi* Macf. × *Poncitrus trifoliata* | 29 | August 2019 | 30 |
| Hendry | US-812 and X-639 | Midsweet | *C. reticulata* ‘Sunki’ *× P. trifoliata* ‘Benecke; *C. reticulata* Blanco ‘Cleopatra’ mandarin *× P. trifoliata* | 10 | May 2018 | 24 |
| Hendry | Carrizo | Midsweet | *C. sinensis* Osbeck × *P. trifoliate* | 11 | August 2018 | 470 |
| Collier | US-802; X-639; Cleopatra | Midsweet | C. *grandis* Osbeck ‘Siamese’ × *P. trifoliata* ‘Gotha Road’; *C. reticulata* Blanco ‘Cleopatra’ mandarin *× P. trifoliata*; *C. reticulata* Blanco | 10 | July 2018 | 70 |

**Table S2.** Summary of the sequence data, including the total number of samples, raw reads, high-quality reads retained after the sequencing analysis, minimum (min) and maximum (max) number of reads per sample, average number of reads per sample, and values of Good’s coverage index for each rhizobiome study.

| Crop | Total n. of samples | Total n. of raw sequence reads | Total n. of high-quality reads | Min, Max- n. of reads per sample | Average n. of reads per sample | Good’s coverage (%) |
| --- | --- | --- | --- | --- | --- | --- |
| Rice | 132 | 5860040 | 2912056 | 15456, 22356 | 17564 | 98.7 ± 1.4 |
| Wheat | 44 | 66345 | 46750 | 941, 1324 | 1062 | 96.1 ± 1.1 |
| Maize | 4866 | 627638736 | 314958688 | 45650, 67569 | 56545 | 93.0 ± 0.7 |
| Citrus #1 | 23 | 640251 | 321625 | 11456, 14236 | 12498 | 95.5 ± 1.4 |
| Citrus #2 | 370 | 11245456 | 5632651 | 12456, 15684 | 13969 | 94.6 ± 1.1 |
| Sugarcane | 99 | 130858200 | 5587586 | 48563, 78656 | 62689 | 97.1 ± 0.8 |
| Tomato | 54 | 1503861 | 721563 | 11456, 14569 | 12478 | 96.3 ± 1.1 |
| Total | 5588 | 777812889 | 330180919 |  |  |  |

**Table S3.** Number of ASVs identified in the taxonomic and functional core rhizobiomes for each rhizobiome study. For each row, values followed by the same letter are not statistically different according to the Welch’s and Benjamini–Hochberg FDR multiple test correction. *p*-values < 0.05 were considered significant.

| Rhizobiome study | Number of ASVs | |
| --- | --- | --- |
|  | Taxonomic core rhizobiome | Functional core rhizobiome |
| Rice | 145^b^ | 678^a^ |
| Wheat | 64^b^ | 538^a^ |
| Maize | 122^b^ | 574^a^ |
| Citrus #1 | 130^b^ | 389^a^ |
| Citrus #2 | 85^b^ | 454^a^ |
| Sugarcane | 366^b^ | 910^a^ |
| Tomato | 420^b^ | 744^a^ |

**Table S4.** Number and taxonomic affiliation of the ASVs shared between the taxonomic and functional core rhizobiomes for rice (A), wheat (B), maize (C), citrus #1 (D), citrus #2 (E), sugarcane (F), and tomato (G).

| Rhizobiome study | Number of shared ASVs | Genus |
| --- | --- | --- |
| Rice | 69 | *Azospirillum* (17 ASVs), *Bradyrhizobium* (10 ASVs), *Streptomyces* (6 ASVs), *Hydrogenophaga* (6 ASVs), *Flavisobacter* (5 ASVs), *Anabaena* (5 ASVs), *Rhodobacter* (4 ASVs), *Propionivibrio* (3 ASVs), *Chthonomonas* (3 ASVs), *Geobacter* (3 ASVs), *Methylomonas* (3 ASVs), *Anaeromuxobacter* (1 ASV), *Rhodoferax* (1 ASV), *Pleomorphomonas* (1 ASV), Dechoromonas (1 ASV) |
| Wheat | 39 | *Bacillus* (8 ASVs), *Flavobacterium* (6 ASVs), *Pseudomonas* (5 ASVs), *Devosia* (5 ASVs), *Acidibacter* (3 ASVs), *Streptomyces* (3 ASVs), *Sphingomonas* (2 ASVs), *Arthrobacter* (2 ASVs), *Lysobacter* (1 ASV), *Microbacterium* (1 ASV), *Nocardioides* (1 ASV), *Pedobacter* (1 ASV), *Promicronospora* (1 ASV) |
| Maize | 57 | *Bradyrhizobium* (12 ASVs), *Agrobacterium* (10 ASVs), *Mesorhizobium* (7 ASVs), *Flavobacterium* (6 ASVs), *Devosia* (4 ASVs), *Pseudomonas* (4 ASVs), *Sphingomonas* (4 ASVs), *Streptomyces* (3 ASVs), *Hypomicrobium* (3 ASVs), *Kaistobacter* (2 ASVs), *Pedobacter* (1 ASV), *Steroidobacter* (1 ASV) |
| Citrus #1 | 54 | *Bradyrhizobium* (9 ASVs), *Burkholderia* (7 ASVs), *Rhizobium* (7 ASVs), *Agrobacterium* (5 ASVs), *Pseudomonas* (3 ASVs), *Cupriavius* (3 ASVs), *Sphingomonas* (3 ASVs), *Achromobacter* (3 ASVs), *Xanthomonas* (2 ASVs), *Ensifer* (2 ASVs), *Chitinophaga* (2 ASVs), *Acidovorax* (2 ASVs), *Acidomonas* (1 ASV), *Azotobacter* (1 ASV), *Cellvibrio* (1 ASV), *Dyadobacter* (1 ASV), *Mesorhizobium* (1 ASV), *Microbacterium* (1 ASV) |
| Citrus #2 | 55 | *Bradyrhizobium* (6 ASVs), *Burkholderia* (4 ASVs), *Xanthomonas* (4 ASVs), *Rhizobium* (4 ASVs), *Streptomyces* (2 ASVs), *Sphingomonas* (2 ASVs), *Pseudomonas* (2 ASVs), *Nitrospira* (2 ASVs), *Ensifer* (2 ASVs), *Cupriavidus* (2 ASVs), *Cellvibrio* (2 ASVs), *Achromobacter* (2 ASVs), *Acidomonas* (2 ASVs), *Acidovorax* (2 ASVs), *Agrobacterium* (2 ASVs), *Azotobacter* (2 ASVs), *Bdellovibrio* (2 ASVs), *Candidatus Nitrososphaera* (1 ASV), *Chitinophaga* (1 ASV), *Devosia* (1 ASV), *Dyadobacter* (1 ASV), *Gaiella* (1 ASVs), *Gemmatimonas* (1 ASVs), *Geodermatophilus* (1 ASV), *Haliangium* (1 ASV), *Iamia* (1 ASV), *Mesorhizobium* (1 ASV), *Microbacterium* (1 ASV), *Mycobacterium* (1 ASV), *Nitrosomas* (1 ASV) |
| Sugarcane | 140 | *Bradyrhizobium* (30 ASVs), *Bacillus* (17 ASVs), *Burkholderia* (17 ASVs), *Streptomyces* (14 ASVs), *Xanthomonas* (10 ASVs), *Pseudomonas* (10 ASVs), *Chitinophaga* (8 ASVs), *Sphingomonas* (8 ASVs), *Serratia* (7 ASVs), *Rhodoplanes* (7 ASVs), *Mycobacterium* (6 ASVs), *Erwinia* (6 ASVs) |
| Tomato | 182 | *Rhizobium* (28 ASVs), *Burkholderia* (19 ASVs), *Acidobacteria* (19 ASVs), *Xanthomonas* (15 ASVs), *Sphingomonas* (17 ASVs), *Pseudomonas* (17 ASVs), *Nitrosomonas* (14 ASVs), *Flavobacterium* (14 ASVs), *Cytophaga* (11 ASVs), *Lysobacter* (10 ASVs), *Myxococcus* (9 ASVs), *Pseudonocaria* (9 ASVs) |

**Table S5.** Number of pathways and KOs identified in taxonomic and functional rhizobiomes for each rhizobiome study. For each row, pathways and KOs followed by the same letter are not statistically different according to the Welch’s and Benjamini–Hochberg FDR multiple test correction. *p*-values < 0.05 were considered significant.

| Rhizobiome study | Number of pathways | | Number of KOs | |
| --- | --- | --- | --- | --- |
|  | Taxonomic core rhizobiome | Functional core rhizobiome | Taxonomic core rhizobiome | Functional core rhizobiome |
| Rice | 49^b^ | 89^a^ | 375^b^ | 1256^a^ |
| Wheat | 35^b^ | 98^a^ | 385^b^ | 825^a^ |
| Maize | 54^b^ | 85^a^ | 420^b^ | 865^a^ |
| Citrus #1 | 39^b^ | 69^a^ | 523^b^ | 1142^a^ |
| Citrus #2 | 48^b^ | 80^a^ | 484^b^ | 854^a^ |
| Sugarcane | 51^b^ | 92^a^ | 451^b^ | 945^a^ |
| Tomato | 39^b^ | 79^a^ | 545^b^ | 989^a^ |

**Table S6.** Hub taxa identified in rice (A), wheat (B), maize (C), citrus #1 (D), citrus #2 (E), sugarcane (F), and tomato (G) rhizobiome co-occurrence networks (Fig. 4; Fig. S4). Hub taxa belonging to the taxonomic core rhizobiome (TCR) or functional core rhizobiome (FCR) rhizobiome are denoted.

A.

| **Crop** | **Sample** | **ASV_ID** | **Taxonomy** | | | | | | **Degree** | **Betweenness centrality** | **Closeness centrality** |
| --- | --- | --- | --- | --- | --- | --- | --- | --- | --- | --- | --- |
|  |  |  | **Phylum** | **Class** | **Order** | **Family** | **Genus** | **Species** |  |  |  |
| Rice | TCR | ASV_81 | Proteobacteria | Alphaproteobacteria | Rhodospirillales | Rhodospirillaceae | *Azospirillum* | Unclassified | 65 | 0.145 | 0.845 |
|  | FCR | ASV_269 | Proteobacteria | Alphaproteobacteria | Rhizobiales | Methylocystaceae | *Pleomorphomonas* | Unclassified | 54 | 0.124 | 0.745 |
|  | FCR | ASV_147 | Proteobacteria | Alphaproteobacteria | Rhodobacterales | Rhodobacteraceae | *Rhodobacter* | Unclassified | 48 | 0.305 | 0.875 |
|  | FCR | ASV_48 | Actinobacteria | Streptomycetales | Steptomycineae | Steptomycetaceae | *Streptomyces* | Unclassified | 49 | 0.304 | 0.897 |

B.

| **Crop** | **Sample** | **ASV_ID** | **Taxonomy** | | | | | | **Degree** | **Betweenness centrality** | **Closeness centrality** |
| --- | --- | --- | --- | --- | --- | --- | --- | --- | --- | --- | --- |
|  |  |  | **Phylum** | **Class** | **Order** | **Family** | **Genus** | **Species** |  |  |  |
| Wheat | TCR | ASV_384 | Actinobacteria | Streptomycetales | Steptomycineae | Steptomycetaceae | *Streptomyces* | Unclassified | 44 | 0.223 | 0.865 |
|  | TCR | ASV_89 | Proteobacteria | Alphaproteobacteria | Sphingomonadales | Sphingomonadaceae | *Sphingomonas* | Unclassified | 45 | 0.194 | 0.897 |
|  | FCR | ASV_365 | Bacteroidetes | Flavobacteria | Flavobacteriaceae | Flavobacteriaceae | *Flavobacterium* | Unclassified | 39 | 0.056 | 0.746 |
|  | FCR | ASV_224 | Proteobacteria | Gammaproteobacteria | Pseudomonadales | Psedomonadaceae | *Pseudomonas* | Unclassified | 29 | 0.198 | 0.689 |
|  | FCR | ASV_78 | Actinobacteria | Actinomycetales | Micrococcineae | Micrococcaceae | *Arthrobacter* | Unclassified | 14 | 0.158 | 0.748 |

C.

| **Crop** | **Sample** | **ASV_ID** | **Taxonomy** | | | | | | **Degree** | **Betweenness centrality** | **Closeness centrality** |
| --- | --- | --- | --- | --- | --- | --- | --- | --- | --- | --- | --- |
|  |  |  | **Phylum** | **Class** | **Order** | **Family** | **Genus** | **Species** |  |  |  |
| Maize | TCR | ASV_9 | Proteobacteria | Alphaproteobacteria | Rhizobiales | Rhizobiaceae | *Agrobacterium* | Unclassified | 4 | 0.145 | 0.789 |
|  | TCR | ASV_78 | Proteobacteria | Alphaproteobacteria | Rhizobiales | Bradyrhizobiaceae | *Bradyrhizobium* | Unclassified | 25 | 0.167 | 0.654 |
|  | FCR | ASV_159 | Proteobacteria | Gammaproteobacteria | Pseudomonadales | Psedomonadaceae | *Pseudomonas* | Unclassified | 55 | 0.154 | 0.856 |
|  | FCR | ASV_248 | Proteobacteria | Alphaproteobacteria | Sphingomonadales | Sphingomonadaceae | *Kaistobacter* | Unclassified | 18 | 0.148 | 0.645 |
|  | FCR | ASV_98 | Actinobacteria | Streptomycetales | Steptomycineae | Steptomycetaceae | *Streptomyces* | Unclassified | 33 | 0.210 | 0.654 |

D.

| **Crop** | **Sample** | **ASV_ID** | **Taxonomy** | | | | | | **Degree** | **Betweenness centrality** | **Closeness centrality** |
| --- | --- | --- | --- | --- | --- | --- | --- | --- | --- | --- | --- |
|  |  |  | **Phylum** | **Class** | **Order** | **Family** | **Genus** | **Species** |  |  |  |
| Citrus #1 | TCR | ASV_258 | Proteobacteria | Betaproteobacteria | Burkholderiales | Burkholderiaceae | *Burkholderia* | Unclassified | 56 | 0.133 | 0.845 |
|  | TCR | ASV_89 | Proteobacteria | Alphaproteobacteria | Rhizobiales | Hyphomicrobiaceae | *Devosia* | Unclassified | 64 | 0.145 | 0.789 |
|  | FCR | ASV_159 | Proteobacteria | Alphaproteobacteria | Rhizobiales | Rhizobiaceae | *Ensifer* | Unclassified | 49 | 0.059 | 0.859 |
|  | FCR | ASV_325 | Proteobacteria | Gammaproteobacteria | Pseudomonadales | Psedomonadaceae | *Pseudomonas* | Unclassified | 69 | 0.165 | 0.945 |
|  | FCR | ASV_57 | Proteobacteria | Alphaproteobacteria | Rhizobiales | Rhizobiaceae | *Rhizobium* | Unclassified | 48 | 0.048 | 0.948 |
|  | FCR | ASV_78 | Proteobacteria | Betaproteobacteria | Burkholderiales | Burkholderiaceae | *Cupriavidus* | Unclassified | 59 | 0.059 | 0.849 |

E.

| **Crop** | **Sample** | **ASV_ID** | **Taxonomy** | | | | | | **Degree** | **Betweenness centrality** | **Closeness centrality** |
| --- | --- | --- | --- | --- | --- | --- | --- | --- | --- | --- | --- |
|  |  |  | **Phylum** | **Class** | **Order** | **Family** | **Genus** | **Species** |  |  |  |
| Citrus #2 | TCR | ASV_59 | Proteobacteria | Gammaproteobacteria | Pseudomonadales | Psedomonadaceae | *Azotobacter* | Unclassified | 39 | 0.032 | 0.541 |
|  | TCR | ASV_64 | Nitrospirae | Nitrospira | Nitrospirales | Nitrospiraceae | *Nitrospira* | Unclassified | 35 | 0.029 | 0.554 |
|  | FCR | ASV_85 | Proteobacteria | Gammaproteobacteria | Xanthomonadales | Xanthomonadales | *Xanthomonas* | Unclassified | 65 | 0.068 | 0.724 |
|  | FCR | ASV_259 | Proteobacteria | Gammaproteobacteria | Pseudomonadales | Psedomonadaceae | *Pseudomonas* | Unclassified | 56 | 0.066 | 0.824 |
|  | FCR | ASV_46 | Proteobacteria | Alphaproteobacteria | Rhizobiales | Rhizobiaceae | *Rhizobium* | Unclassified | 42 | 0.071 | 0.633 |
|  | FCR | ASV_6 | Proteobacteria | Betaproteobacteria | Burkholderiales | Burkholderiaceae | *Burkholderia* | Unclassified | 57 | 0.052 | 0.645 |

F.

| **Crop** | **Sample** | **ASV_ID** | **Taxonomy** | | | | | | **Degree** | **Betweenness centrality** | **Closeness centrality** |
| --- | --- | --- | --- | --- | --- | --- | --- | --- | --- | --- | --- |
|  |  |  | **Phylum** | **Class** | **Order** | **Family** | **Genus** | **Species** |  |  |  |
| Sugarcane | TCR | ASV_87 | Proteobacteria | Betaproteobacteria | Burkholderiales | Oxalobacteraceae | *Burkholderia* | Unclassified | 54 | 0.069 | 0.845 |
|  | TCR | ASV_96 | Bacteroidetes | Chitinophagia | Chitinophagales | Chitinophagaceae | *Chitinophaga* | Unclassified | 49 | 0.058 | 0.654 |
|  | FCR | ASV_189 | Proteobacteria | Alphaproteobacteria | Sphingomonadales | Sphingomonadaceae | *Sphingomonas* | *echinoides* | 45 | 0.068 | 0.845 |
|  | FCR | ASV_320 | Proteobacteria | Alphaproteobacteria | Rhizobiales | Bradyrhizobiaceae | *Bradyrhizobium* | Unclassified | 53 | 0.066 | 0.875 |
|  | FCR | ASV_198 | Proteobacteria | Gammaproteobacteria | Enterobacterales | Yersiniaceae | *Serratia* | Unclassified | 48 | 0.054 | 0.879 |

G.

| **Crop** | **Sample** | **ASV_ID** | **Taxonomy** | | | | | | **Degree** | **Betweenness centrality** | **Closeness centrality** |
| --- | --- | --- | --- | --- | --- | --- | --- | --- | --- | --- | --- |
|  |  |  | **Phylum** | **Class** | **Order** | **Family** | **Genus** | **Species** |  |  |  |
| Tomato | TCR | ASV_158 | Proteobacteria | Gammaproteobacteria | Xanthomonadales | Xanthomonadaceae | *Xanthomonas* | Unclassified | 59 | 0.078 | 0.874 |
|  | FCR | ASV_425 | Proteobacteria | Alphaproteobacteria | Sphingomonadales | Sphingomonadaceae | *Sphingomonas* | Unclassified | 45 | 0.056 | 0.856 |
|  | FCR | ASV_84 | Proteobacteria | Betaproteobacteria | Nitrosomonadales | Nitrosomonadaceae | *Nitrosomonas* | Unclassified | 54 | 0.088 | 0.877 |
|  | FCR | ASV_111 | Proteobacteria | Betaproteobacteria | Burkholderiales | Oxalobacteraceae | *Burkholderia* | Unclassified | 52 | 0.054 | 0.912 |

**References**

1. Apprill, A. *et al*. Minor revision to V4 region SSU rRNA 806R gene primer greatly increases detection of SAR11 bacterioplankton. *Aquat Microb. Ecol.* **75**, 129–137 (2015).

2. Hamonts, K. *et al*. Field study reveals core plant microbiota and relative importance of their drivers. *Environ. Microbiol.* **20**, 124–140 (2018).
